# Nuclear HMGB1 protects from nonalcoholic fatty liver disease through negative regulation of liver X receptor

**DOI:** 10.1126/sciadv.abg9055

**Published:** 2022-03-25

**Authors:** Jean Personnaz, Enzo Piccolo, Alizée Dortignac, Jason S. Iacovoni, Jérôme Mariette, Vincent Rocher, Arnaud Polizzi, Aurélie Batut, Simon Deleruyelle, Lucas Bourdens, Océane Delos, Lucie Combes-Soia, Romain Paccoud, Elsa Moreau, Frédéric Martins, Thomas Clouaire, Fadila Benhamed, Alexandra Montagner, Walter Wahli, Robert F. Schwabe, Armelle Yart, Isabelle Castan-Laurell, Justine Bertrand-Michel, Odile Burlet-Schiltz, Catherine Postic, Pierre-Damien Denechaud, Cédric Moro, Gaelle Legube, Chih-Hao Lee, Hervé Guillou, Philippe Valet, Cédric Dray, Jean-Philippe Pradère

**Affiliations:** 1 Institut RESTORE, UMR 1301, Institut National de la Santé et de la Recherche Médicale (INSERM), CNRS-Université Paul Sabatier, Université de Toulouse, Toulouse, France.; 2 Institut des Maladies Métaboliques et Cardiovasculaires, UMR 1297/I2MC, Institut National de la Santé et de la Recherche Médicale (INSERM), Université de Toulouse, Toulouse, France.; 3 MIAT, Université de Toulouse, INRAE, 31326 Castanet-Tolosan, France.; 4 Molecular, Cellular, and Developmental Biology Unit (MCD), Centre de Biologie Intégrative (CBI), UPS, CNRS, Toulouse, France.; 5 Toxalim, INRAE UMR 1331, ENVT, INP-Purpan, University of Toulouse, Paul Sabatier University, F-31027, Toulouse, France.; 6 MetaToul-MetaboHUB, Toulouse, France.; 7 Institut de Pharmacologie et de Biologie Structurale, IPBS, Université de Toulouse, CNRS, UPS, Toulouse, France.; 8 Plateforme GeT, Genotoul, 31100 Toulouse, France.; 9 Université de Paris, Institut Cochin, CNRS, INSERM, F- 75014 Paris, France.; 10 Center for Integrative Genomics, University of Lausanne, Le Génopode, CH-1015 Lausanne, Switzerland.; 11 Lee Kong Chian School of Medicine, Nanyang Technological University Singapore, Clinical Sciences Building, 11 Mandalay Road, Singapore 308232, Singapore.; 12 Department of Medicine, Columbia University, New York, NY, USA.; 13 Department of Molecular Metabolism, Harvard T.H. Chan School of Public Health, Boston, MA, USA.

## Abstract

Dysregulations of lipid metabolism in the liver may trigger steatosis progression, leading to potentially severe clinical consequences such as nonalcoholic fatty liver diseases (NAFLDs). Molecular mechanisms underlying liver lipogenesis are very complex and fine-tuned by chromatin dynamics and multiple key transcription factors. Here, we demonstrate that the nuclear factor HMGB1 acts as a strong repressor of liver lipogenesis. Mice with liver-specific *Hmgb1* deficiency display exacerbated liver steatosis, while *Hmgb1*-overexpressing mice exhibited a protection from fatty liver progression when subjected to nutritional stress. Global transcriptome and functional analysis revealed that the deletion of *Hmgb1* gene enhances LXRα and PPARγ activity. HMGB1 repression is not mediated through nucleosome landscape reorganization but rather via a preferential DNA occupation in a region carrying genes regulated by LXRα and PPARγ. Together, these findings suggest that hepatocellular HMGB1 protects from liver steatosis development. HMGB1 may constitute a new attractive option to therapeutically target the LXRα-PPARγ axis during NAFLD.

## Teaser

Nuclear form of HMGB1 in hepatocytes protects from liver steatosis development.

## INTRODUCTION

Along the epidemic of obesity, nonalcoholic fatty liver disease (NAFLD) is progressing worldwide, affecting nearly 25% of the worldwide adult population (*1*) and generating numerous complications such as liver insulin resistance, nonalcoholic steatohepatitis, and hepatocellular carcinoma (*2*). Liver steatosis consists of ectopic lipid storage within the hepatocytes, which aims at buffering circulating lipids and thus preventing lipotoxicity in different organs. Mechanisms underlying lipogenesis (from lipid uptake to lipid esterification and de novo lipogenesis) are extremely complex and consist of a subtle orchestration of the actions of different transcription factors (TFs) in close coordination with chromatin dynamics (*3*).

Among TFs involved in liver lipogenesis regulation, liver X receptors (LXRs) are members of the nuclear hormone receptor superfamily and are among the most central/dominant actors in this process. LXRs consist of two isotypes that share a very high homology but differ in their tissue expression profile. While LXRα (NR1H3) is mainly expressed in metabolic tissues (liver and adipose tissues), LXRβ (NR1H2) is expressed ubiquitously (*4*). In the context of dyslipidemia or fasting/refeeding conditions and after activation by certain lipid species (*5*), LXRs directly coordinate, in a duo with its obligate partner, retinoic acid receptor (RXR), the expression of numerous key enzymes involved in cholesterol and lipid metabolism (*Abcg5*, *Abcg8*, *Fasn*, and *Scd-1*), but are also capable to modulate indirectly the lipogenesis through the regulation of other key TFs like SREBP1c, ChREBP, or PPARγ (*4*, *6*, *7*) that are also involved in the lipogenic transcription program. The current consensus on liver lipogenesis is that there is a hierarchical interplay between all TFs involved, where LXR is a very central piece; SREBP1c and ChREBP are crucial downstream key players, while PPARγ’s role appears more supportive (*3*). LXR activity is subtly regulated by the interaction with the nuclear receptor corepressors (NCoRs) or the nuclear receptor coactivator protein complex (*3*) upon specific agonist activation. Recent evidences are now showing the emerging role of some methylase/demethylase enzymes in the modulation of LXR activity through the chromatin packaging and subsequent availability, adding one more complex layer of regulation (*8*, *9*). Global knockout of LXRs induces a severe reduction of liver lipid synthesis in wild-type mice and could even prevent liver steatosis in ob/ob mice (*10*–*12*). LXRα deletion knockout leads to a down-regulation of *Srebf1* expression associated with a reduced lipogenesis (*6*). Moreover, LXR agonist treatment increases plasma and hepatic TG (triglyceride) in mice and humans (*13*, *14*), supporting a key role of LXRs in fatty acid synthesis and liver steatosis progression. Therapeutic targeting of LXRs is still challenging, as adverse effects have been described (*14*) and more insights regarding LXR upstream regulators may be helpful to design novel therapeutic avenues. Among other pharmacological strategies, targeting PPARs sparks a lot of attention, and the efficacy of several synthetic agonists is currently tested in clinical trials (*15*). PPARs, similarly to LXR, are nuclear receptors, gathering several subfamilies (α, β/δ, and γ) (*16*). In the hepatocyte, the lipogenic activity is supported by PPARγ, but its activity at the basal state is rather negligible. Its involvement as a trigger during hepatic lipogenesis is still discussed. The role of PPARγ per se in high-fat diet (HFD)–induced hepatosteatosis is supported by one study (*17*), while several other reports demonstrated its important contribution on morbid backgrounds such as ob/ob or A-ZIP/F-1 mice (*18*, *19*). PPARγ is therefore not envisioned as key triggering element but rather as an important relay in the lipogenic response (*20*). Together, the current consensus on liver lipogenesis is that there is a hierarchical interplay between all TFs involved, where LXR is a very potent piece and PPARγ is a more supportive player. However, the upstream regulators/factors that determine this complex organization are not well understood.

HMGB1 belongs to the family of high-mobility group proteins, which after the histones represents the most abundant proteins in the nucleus. In recent years, HMGB1 has also been scrutinized for its role in the extracellular compartment as a potent inflammatory factor, notably during sterile inflammation (*18*, *21*). Originally, however, HMGB1 has been known for its role in the nucleus (*22*) as a protein capable of binding chromatin on unspecific domains (*23*) in a very dynamic manner (*24*). HMGB1 may affect several biological functions such as VDJ recombination, DNA repair (*25*), chromatin assembly, and gene transcription through different mechanisms, such as DNA bending/looping, nucleosome formation (*26*, *27*), and interaction with the transcription machinery including TFs themselves (*24*, *28*–*30*). A very recent report depicts nuclear HMGB1 as an even more versatile factor able to bind to topologically associated domains or RNA directly to regulate proliferation or senescence programs (*31*). In cultured cells, while HMGB1 deletion leads to minor changes in histone numbers, it results in notable changes of the RNA pool (*27*), in local chromatin remodeling (*32*) or the global transcriptome (*31*). However, only a sparse number of studies have been carried out in vivo (*32*). The global ablation of *Hmgb1* generates a severe phenotype with perinatal mortality (*33*), likely due to a defective glucocorticoid signaling leading to a poor utilization of hepatic glycogen and resulting in a lethal hypoglycemia, whereas hepatocyte-specific HMGB1 ablation did not have a major impact under homeostatic conditions (*34*). Thus, in this context, it seems particularly relevant to explore the role of nuclear HMGB1 in vivo especially during metabolic stress, where the dynamics of the chromatin are critical to orchestrate the activity of key TFs and gene transcription programs to buffer stress mediators and maintain whole-body homeostasis.

Here, we unveiled the important role of HMGB1 in the repressive effect of the LXRα/PPARγ axis, during metabolic stress, as demonstrated by increased liver steatosis in hepatocyte-specific *Hmgb1* knockout (HMGB1^ΔHep^) mice and a reduced hepatic lipid load in *Hmgb1*-overexpressing mice when subjected to nutritional stress. In vitro assays further confirmed the repressive action that HMGB1 exerts on LXRα activity specifically. Together, our data reveal a novel role of HMGB1 in alleviating liver steatosis through the repression of the LXRα/PPARγ axis during metabolic stress.

## RESULTS

### Hepatic deletion of Hmgb1 increases liver steatosis during metabolic stress

**Fig. 1.**
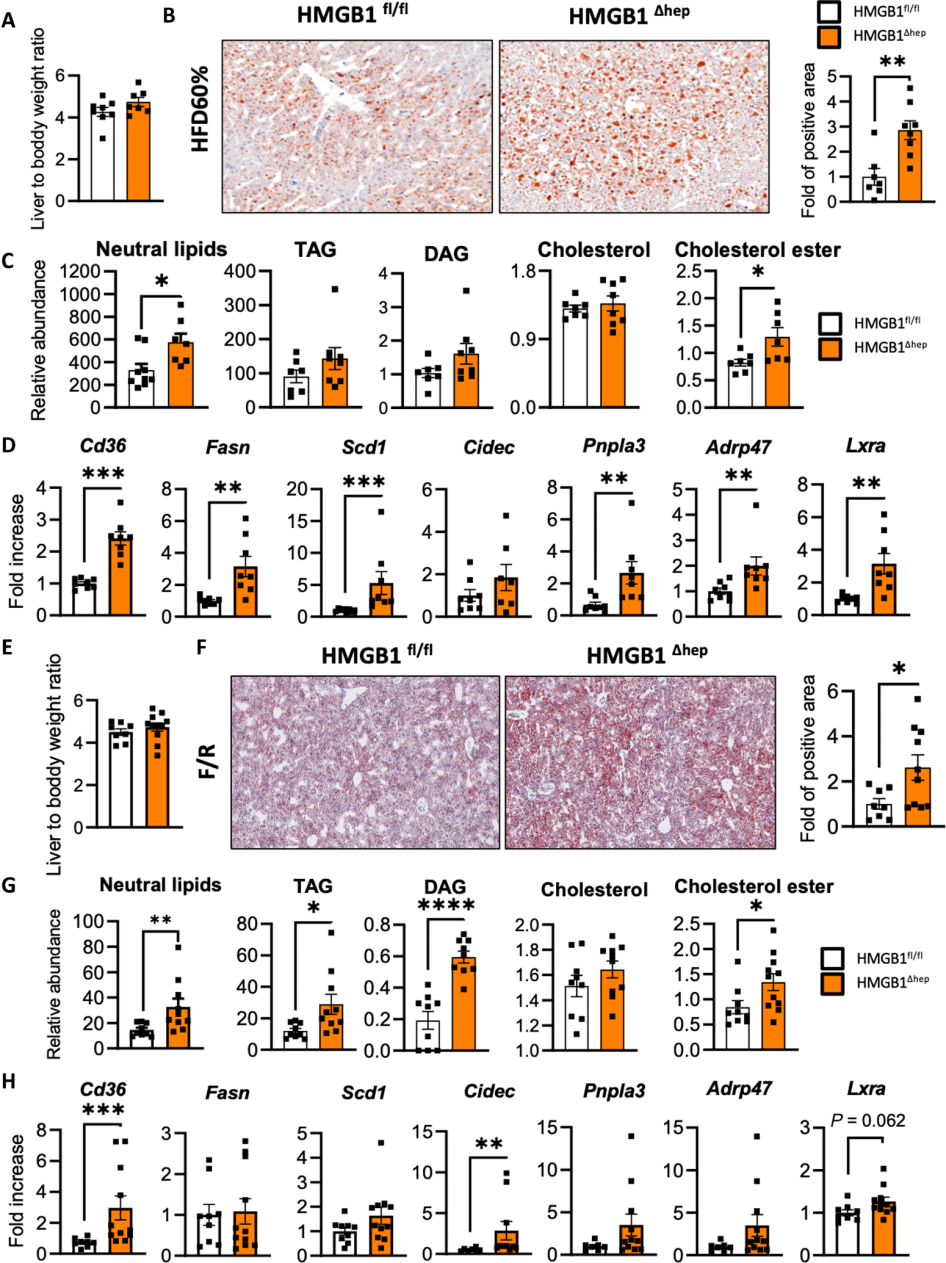
Hepatocyte-specific *Hmgb1*-deleted mice on HFD or after fasting/refeeding challenge exhibit severe liver steatosis. (**A**) Liver/body weight ratio, (**B**) Oil Red O staining on liver section with quantification, (**C**) neutral lipid analysis, and (**D**) mRNA expression of hepatic steatosis markers from liver biopsies of HMGB1^fl/fl^ and HMGB1^ΔHep^ mice subjected to 12-week HFD. (**E**) Liver/body weight ratio, (**F**) Oil Red O staining on liver section with quantification, (**G**) neutral lipid analysis, and (**H**) mRNA expression of hepatic steatosis markers from liver biopsies of HMGB1^fl/fl^ and HMGB1^ΔHep^ mice after a fasting/refeeding challenge. Data are means ± SEM from *n* = 7 (HMGB1^fl/fl^) or *n* = 8 (HMGB1^ΔHep^) per group for the HFD protocol (A to D) and from *n* = 8 (HMGB1^fl/fl^) or *n* = 8 (HMGB1^ΔHep^) per group for the F/R protocol (E to H). *P < 0.05, **P < 0.01, ***P < 0.001, and ****P < 0.0001 by unpaired Mann-Whitney comparison.

*Hmgb1* hepatocyte-specific knockout mice (HMGB1^ΔHep^) under chow diet (CD) feeding display no major changes in liver transcriptome and no drastic phenotype of glycogen utilization compared to control mice (HMGB1^fl/fl^) (*34*), contrasting findings from the global *Hmgb1* knockout on metabolism, possibly due to particular functions during development (*33*). This prompted us to clarify the precise function of HMGB1 in liver metabolism by studying the role of HMGB1 as a potential regulator of global and/or hepatic energy metabolism in adult mice using a careful characterization of HMGB1^fl/fl^ and HMGB1^ΔHep^ mice subjected to metabolic stress. A complete metabolic checkup in adult mice upon CD showed that successful deletion of *Hmgb1* gene in liver lysates (fig. S1, A to C) did not affect circulating levels of HMGB1 (fig. S1D), serum liver enzyme levels (fig. S1E), body weight (fig. S1F), lean/fat mass ratio (fig. S1G), fasting blood glucose levels, and glucose homeostasis (fig. S1H) nor generated any changes in hepatic lipid contents (fig. S1I). However, a high-throughput real-time quantitative polymerase chain reaction (RT-qPCR) gene expression profiling targeting metabolic pathways revealed that many key genes involved in lipid metabolism and lipogenesis, such as *Cd36*, *Fasn*, *Acaca*, or *Acly*, were significantly up-regulated in the liver of HMGB1^ΔHep^ mice compared to HMGB1^fl/fl^ mice (fig. S1J). In addition, using an immunoblot, the up-regulation of key lipogenic enzymes has been confirmed at the protein level in *Hmgb1*-null livers (fig. S1K). Collectively, these data suggest that, while supporting conclusions from a previous report (*34*) on the minor role of HMGB1 in systemic and liver metabolic homeostasis, its function might become more relevant in the setting of metabolic stress. To test this hypothesis, HMGB1^fl/fl^ and HMGB1^ΔHep^ mice were subjected to an HFD feeding (HFD60%). After 12 weeks of this regimen, HMGB1^fl/fl^ control mice showed the expected weight gain and glucose metabolism deterioration compared to mice fed CD (not shown). In this context, after HFD60%, *Hmgb1* gene deletion was still efficient, as shown in liver sections and nuclear extracts (fig. S2, A and B). Both genotypes displayed similar weight gain (fig. S2C) and similar fat mass (fig. S2D) and shared identical physiological parameters (food intake, respiratory quotient, and physical activity) (fig. S2, E to G). However, HMGB1^ΔHep^ mice exhibited a slight upward trend for the liver/body weight ratio (Fig. 1A) and significant increases in Oil Red O staining (Fig. 1B) and in liver lipid content, especially for global neutral lipids and cholesterol ester, compared to control mice (Fig. 1C). In addition, mRNA expression analysis revealed an up-regulation of key genes and proteins involved in liver lipid metabolism and lipogenesis (Fig. 1D and fig. S2H) in livers from HMGB1^ΔHep^ mice compared to control littermates. To further challenge the lipogenic pathway using a more acute nutritional setting without confounding effects related to a 12-week HFD, HMGB1^fl/fl^ and HMGB1^ΔHep^ mice were subjected to a 6-hour fast and an 8-hour CD refeeding (F/R) experiment. Similar to HFD, *Hmgb1* gene ablation was still significant (fig. S2, I and J). Hepatic lipid accumulation in HMGB1^ΔHep^ mice was more pronounced compared to control mice, as supported by a trend toward a higher liver/body weight ratio (Fig. 1E) and a marked increase of Oil Red O staining on liver sections (Fig. 1F), hepatic lipid levels (Fig. 1G), and lipogenic gene expression and protein levels (Fig. 1H and fig. S2K) in liver biopsies from HMGB1^ΔHep^ mice compared to HMGB1^fl/fl^ mice. To confirm the HMGB1^ΔHep^ mice phenotype, several other diets designed to challenge the hepatic lipogenesis were implemented, such as 24-week HFD, 8-week choline-deficient HFD, and a 12-week high-fat high-fructose diet, all showing a consistent and more pronounced liver steatosis in HMGB1^ΔHep^ mice compared to HMGB1^fl/fl^ mice (not shown). These results indicate that under several steatosis-promoting regimens, *Hmgb1* deletion in hepatocytes is associated with a more active liver lipogenesis, suggesting that HMGB1 might play a repressive role on liver lipid synthesis, thereby preventing steatosis.

### Hepatic HMGB1 overexpression reduces fatty liver progression

**Fig. 2.**
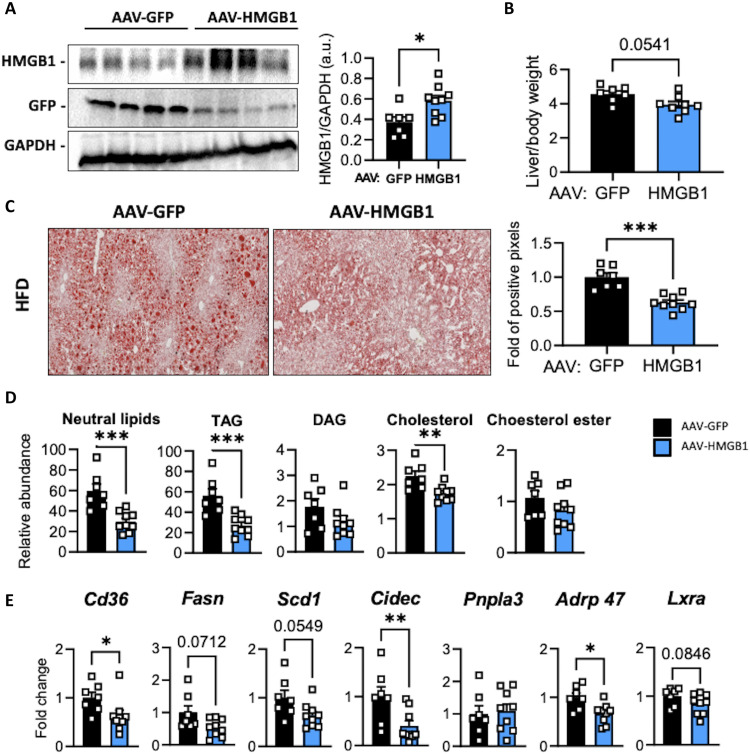
Hepatic *Hmgb1* overexpression decreases liver steatosis in mice subjected to HFD. C57Bl6 mice were infected with either associated adenovirus expressing the eGFP (AAV8-CMV-GFP, *n* = 7) or *Hmgb1* (AAV8-CMV-HMGB1, *n* = 9) sequence and then subjected to 12-week HFD regimen. (**A**) Immunoblot targeting HMGB1 and GFP in liver extracts. GAPDH was used as loading control. (**B**) Liver/body weight ratio. (**C**) Liver steatosis was determined by Oil Red O staining on liver sections, with the quantitative representation displayed on the right. (**D** and **E**) Neutral lipid analysis determined by MS (D) and mRNA expression of hepatic steatosis markers analyzed by RT-qPCR (E). *P < 0.05, **P < 0.01, ***P < 0.001, and ****P < 0.0001 by unpaired Mann-Whitney comparison. a.u., arbitrary units.

To further explore the role of HMGB1 on liver lipid synthesis, and firmly establish HMGB1 as a potent candidate to repress liver steatosis, we set up a gain-of-function model where HMGB1 is overexpressed in vivo using adeno-associated virus (AAV)–mediated gene transfer. To achieve HMGB1 overexpression, we used AAV vector serotype 8 (AAV8), known for its high tropism for hepatocytes, combined with the cytomegalovirus (CMV) promoter, known for its potent activity. Wild-type C57Bl6 mice were injected retro-orbitally with a control AAV8 coding for the enhanced green fluorescent protein (eGFP) (AAV8-CMV-eGFP) or AAV8 coding for HMGB1 (AAV8-CMV-HMGB1) and subjected to HFD60% for 12 weeks. Compared to AAV-GFP mice group, AAV-HMGB1 mice displayed a twofold increase of HMGB1 protein levels in the liver (Fig. 2A) and a lower liver/body weight ratio (Fig. 2B). This is associated with glucose homeostasis improvements supported by a reduced starved glycemia and a better glucose tolerance test (fig. S3, A and B). In addition, hepatic lipid accumulation in AAV8-HMGB1 mice was more reduced compared to control AAV8-eGFP mice (Fig. 2, C to E), as supported by a decrease of Oil Red O staining on liver sections (Fig. 2C), a decrease of hepatic lipid content (Fig. 2D), and a down-regulation in gene expression of liver steatosis canonical markers (*Cd36*, *Cidec*, or *Adrp47*) (Fig. 2E). Together, these data indicated that HMGB1 protein overexpression inhibits fatty liver progression, confirming that HMGB1 represses liver lipid synthesis in vivo.

### Nuclear HMGB1 represses hepatocyte lipogenesis in vivo and in vitro in a cell-autonomous manner

**Fig. 3.**
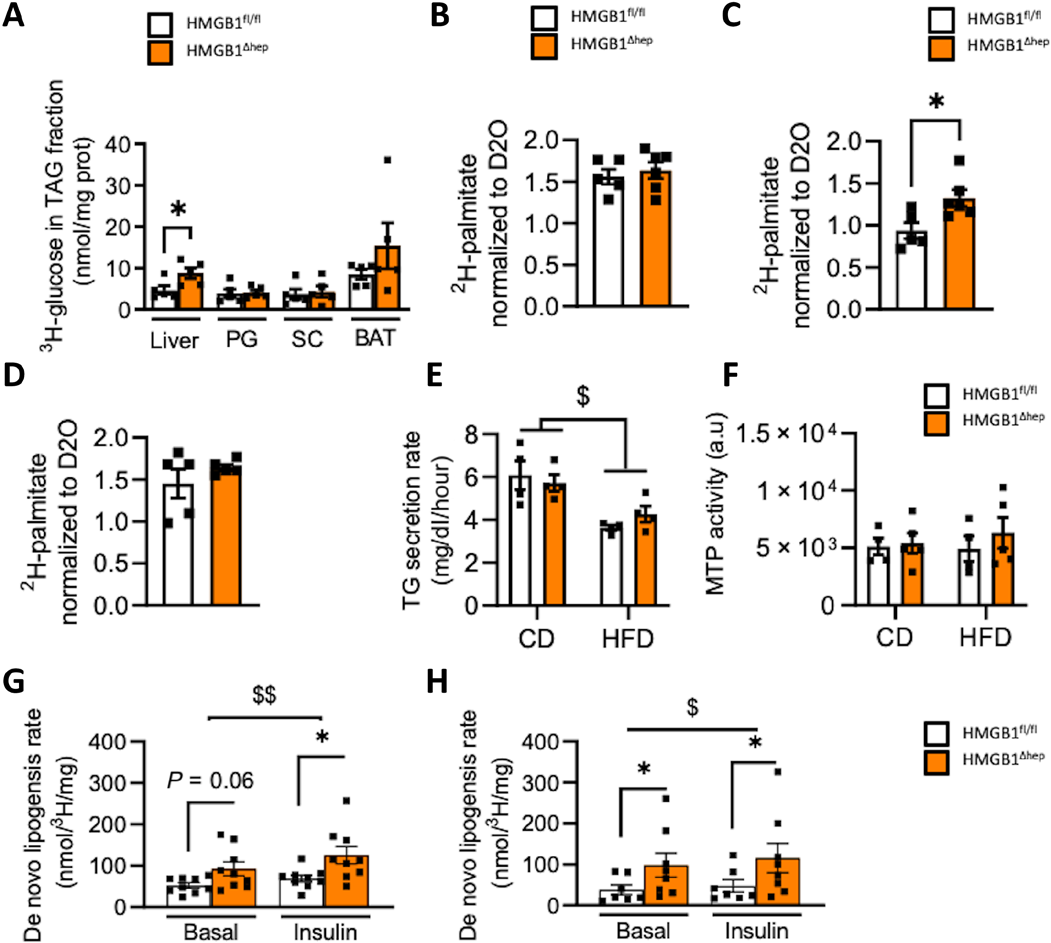
*Hmgb1* deletion increases hepatocyte lipid synthesis in vitro and in vivo. (**A**) In vivo, lipogenesis was measured on HMGB1^fl/fl^ (*n* = 5) and HMGB1^ΔHep^ (*n* = 5) mice. Mice were food-deprived for 6 hours and then injected with ^3^H-glucose (0.4 μCi/g, intraperitoneally) and euthanized 1 hour later, and ^3^H was measured in TG fraction of liver and adipose tissues [perigonadial adipose tissue (PG), subcutaneous adipose tissue (SC), and BAT]. (**B** to **D**) HMGB1^fl/fl^ (*n* = 5) and HMGB1^ΔHep^ (*n* = 5) mice were treated for 3 weeks with D20 in the drinking water (4%), under CD (B), upon HFD60% (C), and after F/R challenge (D). Liver lipogenesis was determined by the amount of ^2^H incorporated in palmitate normalized by ^2^H-labeled plasmatic water. (**E** and **F**) In vivo, assessment of liver lipoprotein secretion was determined by measuring circulating triacylglycerol concentration (*n* = 4 per genotype and diet) (E) and liver MTP activity (F), HMGB1^fl/fl^ (*n* = 4) and HMGB1^ΔHep^ (*n* = 5). (**G** and **H**) Lipid synthesis was measured in vitro, on primary hepatocytes isolated from adult HMGB1^fl/fl^ (*n* = 7 to 9) and HMGB1^ΔHep^ (*n* = 8 to 9) mice on CD (G) and HFD (H). Data are means ± SEM of three independent experiments. *P < 0.05 by unpaired Mann-Whitney comparison or two-way ANOVA. ^$^*P* < 0.05 and ^$$^*P* < 0.01, for treatment effect by one-way ANOVA.

The enhanced hepatosteatosis in HMGB1^ΔHep^ mice may result from an increased activity of lipogenesis in the hepatocytes. To address this question, hepatic lipid synthesis was first monitored in vivo using radiolabeled substrate upon a fasting-refeeding challenge (Fig. 3A). After 6 hours of fasting, HMGB1^fl/fl^ and HMGB1^ΔHep^ mice received a bolus of ^3^H-glucose, and the ^3^H radioisotope incorporation was quantified in the lipid fractions of several tissues after 8 hours of refeeding. Upon CD, while F/R induced a strong ^3^H incorporation mainly in brown adipose tissue (BAT) and liver of HMGB1^fl/fl^ mice (Fig. 3A), this effect was even more pronounced in HMGB1^ΔHep^ mice (Fig. 3A). To further strengthen the conclusions drawn from the ^3^H-glucose strategy, which is sensitive but has some limitations in terms of tracing, we use a complementary approach using hydrogen isotope tracer (^2^H_2_O). HMGB1^fl/fl^ and HMGB1^ΔHep^ mice under CD, subjected to HFD or to F/R, were provided access to ^2^H_2_O-labeled drinking water (4%) 3 weeks before euthanasia (Fig. 3, B to D). The liver lipogenesis was determined by the amount of ^2^H incorporated in palmitate from the triglyceride pool extracted from the liver and normalized by plasmatic water D_2_O enrichment. Upon CD, the percentage of newly made palmitate labeled with ^2^H is comparable in livers from HMGB1^fl/fl^ and HMGB1^ΔHep^ mice. However, during HFD, the percentage of newly made ^2^H palmitate is higher in livers from HMGB1^ΔHep^ mice compared to HMGB1^fl/fl^ mice. After F/R challenge, there is only an upward trend of lipogenesis in HMGB1^ΔHep^ mice compared to wild-type littermates (Fig. 3, B to D). Together, combining ^3^H-glucose and ^2^H_2_O tracers in vivo, these results indicate a higher capacity of *Hmgb1*-null mice to synthesize lipids in the liver during HFD60% or after F/R. In parallel, we evaluated in vivo a potential disturbance of lipoprotein metabolism in HMGB1^ΔHep^ mice upon CD and HFD. The very-low-density lipoprotein (VLDL) secretion after treatment with the lipoprotein lipase inhibitor tyloxapol (Fig. 3E) and the activity of the microsomal triglyceride transfer protein (MTP), a key enzyme involved in lipid export (Fig. 3F), were both identical in HMGB1^fl/fl^ and HMGB1^ΔHep^ mice subjected to CD and HFD, ruling out a possible default of lipid export as a cause of a higher hepatic lipid load in HMGB1^ΔHep^ mice. Present knowledge indicates that the regulation of hepatic lipogenesis depends on the interplay, within the liver, between hepatocytes and nonparenchymal cells and is also influenced by other tissues, mainly the adipose tissue. Therefore, we interrogated whether the increase of liver lipogenesis in HMGB1^ΔHep^ mice could be cell autonomous. To address this point, primary hepatocytes were isolated from HMGB1^fl/fl^ and HMGB1^ΔHep^ mice, and lipogenic activity was assessed in vitro. Consistent with the in vivo data, after isolation from mice under CD, cultured HMGB1^ΔHep^ hepatocytes displayed an increased lipogenic activity compared to HMGB1^fl/fl^ hepatocytes (Fig. 3G). However, lipogenesis was stimulated to the same extent by insulin (Fig. 3G) in hepatocytes from both genotypes. When isolated from HFD-fed mice, HMGB1^ΔHep^ hepatocytes still exhibited a higher lipogenic activity compared to HMGB1^fl/fl^ hepatocytes (Fig. 3H) and insulin slightly increased the lipogenesis independently of the genotypes. Collectively, these results suggest that HMGB1 is repressing lipogenesis in hepatocytes in a cell-autonomous manner.

### Hepatic deletion of Hmgb1 affects specifically liver insulin sensitivity

Studies have reported a correlation between hepatic lipid accumulation and a decreased insulin sensitivity in the liver (*35*). Therefore, we next monitored whether the liver steatosis induced by hepatocyte *Hmgb1* deletion has any effect on glucose homeostasis and/or insulin signaling in mice subjected to HFD60%. Upon HFD, both HMGB1^fl/fl^ and HMGB1^ΔHep^ displayed a similar glucose homeostasis and global insulin sensitivity (fig. S4, A to C). Of note, insulin levels either after starvation or after a bolus of glucose were similar between both groups (fig. S4B). HMGB1^ΔHep^ mice displayed a higher glycemia after 16-hour starvation (fig. S4D), corroborated by a higher area under the curve (AUC) during a pyruvate tolerance test (PyrTT) compared to HMGB1^fl/fl^ mice (fig. S4E) when expressed in absolute values, while no difference was found in neoglucogenic capacity when analyzed as the percentage change from the baseline (fig. S4F). In addition, liver glycogen content, a reflection of insulin sensitivity, was lower in HMGB1^ΔHep^ mice as shown by the periodic acid–Schiff (PAS) coloration (fig. S4, G and H), supporting a compromised glycogen synthesis. Together, these data show that the increased hepatosteatosis in HMGB1^ΔHep^ mice might be associated with a noticeable perturbation of insulin signaling in the liver. This was confirmed by the lower level of AKT phosphorylation already at fed state in the liver of HMGB1^ΔHep^ mice subjected to a 12-week HFD compared to HMGB1^fl/fl^ mice (fig. S4I). To functionally test a possible alteration of insulin sensitivity, HMGB1^fl/fl^ and HMGB1^ΔHep^ mice subjected to CD or a long-term HFD were challenged with an acute injection of insulin (0.75 U/kg) or phosphate-buffered saline (PBS) (figs. S2L and S4J). In CD-fed mice of both genotypes, we observed no differences in the insulin-induced phosphorylation of AKT compared to saline conditions (fig. S2L). In HFD-fed mice, insulin injection induced the phosphorylation of AKT in the liver, adipose tissue (perigonadal fat pad), and skeletal muscle (gastrocnemius) (fig. S4J) in control mice, but the amount of phospho-AKT was lower selectively in liver samples harvested from HMGB1^ΔHep^ mice compared to skeletal muscle and adipose tissue (fig. S4J). Collectively, these data show a selective impact of hepatocellular HMGB1 deficiency on liver insulin signaling upon long-term HFD feeding.

### The signaling of LXR is enhanced in the absence of Hmgb1

**Fig. 4.**
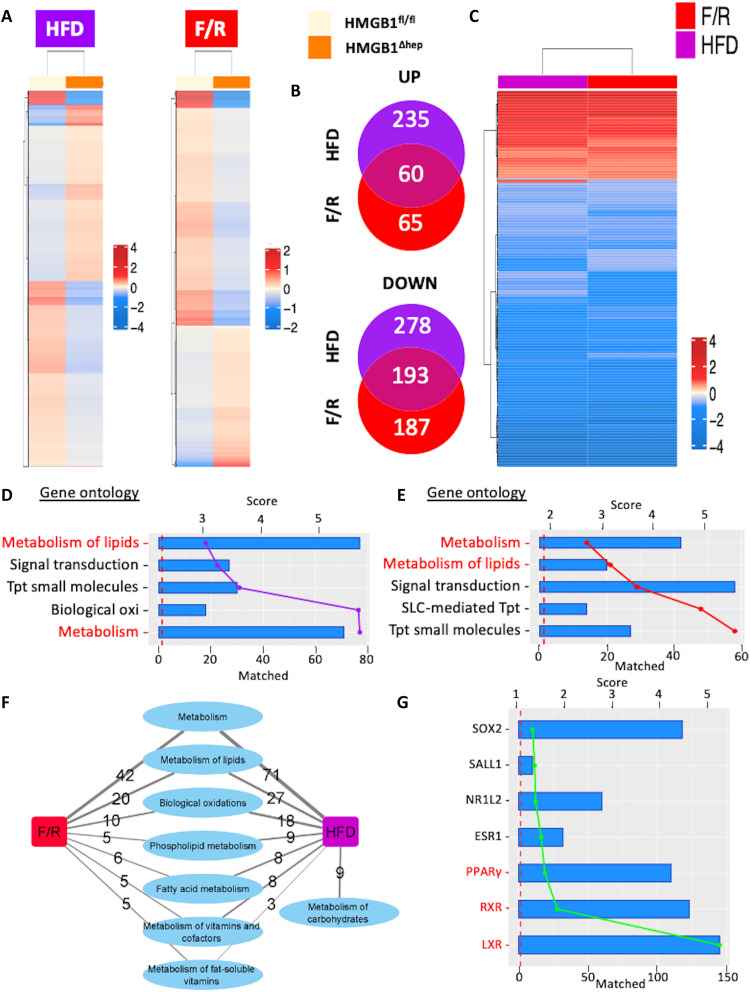
Microarray analysis of hepatic gene expression profiles in HMGB1^ΔHep^ mice. (**A**) Heatmap showing genes that are differentially expressed in the livers of HMGB1^ΔHep^ mice compared to HMGB1^fl/fl^ mice (fold change > 1.5; *P* ≤ 0.01) after HFD (left) or F/R (right). Heatmaps display the mean normalized expression per genotype per nutritional challenge. (**B**) Venn diagram displaying overlap between up- and down-regulated genes in the two regimens. (**C**) Heatmap displaying only differentially expressed genes commonly found in both regimens (fold change > 1.5; *P* ≤ 0.01). (**D** and **E**) Top 5 GO biological processes enriched using gene sets for each regimen, with the −log_10_(*P* value) of enrichment shown as bars and the number of matched genes as colored lines. (**F**) Network displaying Reactome pathways related to metabolism that are enriched by our HMGB1 gene sets from both nutritional challenges. Edge thickness represents the number of genes regulated by HMGB1 among each subcategory. (**G**) Top upstream regulators identified using the ChEA database, with the −log_10_(*P* value) of enrichment as bars and the number of matched genes as the green line. Data are means ± SEM from *n* = 4 (HMGB1^fl/fl^) or *n* = 4 (HMGB1^ΔHep^) per group for the 12-week HFD protocol and from *n* = 4 (HMGB1^fl/fl^) or *n* = 4 (HMGB1^ΔHep^) per group for the F/R protocol.

To unveil the signaling pathways regulated by HMGB1, we performed gene expression profiling using complementary DNA (cDNA) microarray of HMGB1^fl/fl^ and HMGB1^ΔHep^ liver samples from mice subjected to a 12-week HFD regimen or an F/R challenge (Fig. 4). Microarray analysis and unsupervised clustering displayed on the heatmaps showed that deletion of *Hmgb1* caused changes in the liver transcriptome (Fig. 4A). Venn diagrams revealed that in liver samples from HMGB1^ΔHep^ mice, there were 295 up-regulated and 471 down-regulated genes upon HFD and 125 up-regulated and 380 down-regulated genes after F/R (Fig. 4B). Of note, as displayed in the Venn diagram (Fig. 4B), 253 genes (roughly 25%) of the identified genes are similarly regulated in both challenges (HFD and F/R). Hierarchical clustering method showed that most of these genes are subjected to the same type of variations in both conditions (Fig. 4C), suggesting that these groups of genes belong to pathways under robust regulation by *Hmgb1*. The enrichment analysis of these 253 common genes, using the EnrichR database, indicated that among all gene ontology (GO) terms represented in HMGB1^ΔHep^ livers, the most enriched GO terms were “metabolism of lipids” and “metabolism” (Fig. 4, D and E), confirming our histological findings. On the basis of the analysis of the gene network using the Reactome database, numerous genes regulated by HMGB1 in both nutritional conditions are connected to metabolism functions and, more specifically, to lipid metabolism (Fig. 4F). We then narrowed our focus on gene clusters involved in these identified GO terms and further performed analysis on potential upstream regulators involved using EnrichR database (Fig. 4G). Among the identified TFs, LXR, RXR, and PPARγ came up with the highest score. LXRα and PPARγ are well known for their pro-lipogenic activity in the liver, which is in line with the phenotype displayed by the HMGB1^ΔHep^ mice (Fig. 1).

Collectively, our unbiased transcriptomic study indicated that in the liver upon metabolic stress, HMGB1 might repress the expression of gene clusters partly controlled by LXRα and PPARγ and involved in hepatic lipid synthesis.

### Exaggerated hepatic steatosis in the *Hmgb1*-null liver is dependent of LXRα and PPARγ activities

**Fig. 5.**
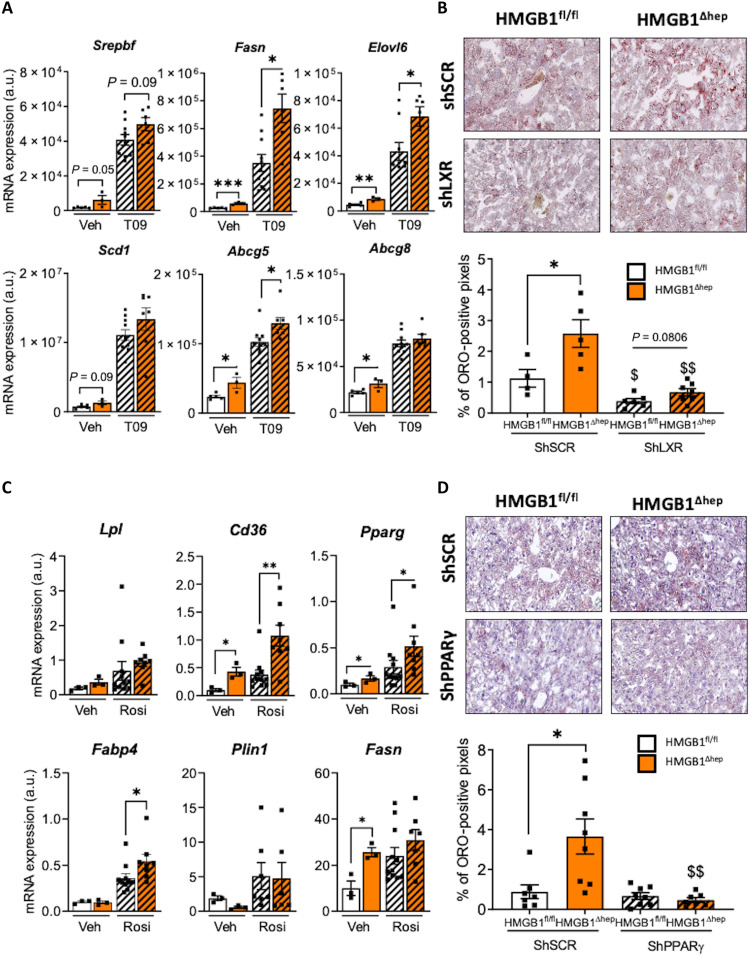
In vivo knockdown of LXR and *PPAR*γ normalizes liver steatosis in HMGB1^ΔHep^ mice. (**A** and **B**) HMGB1^fl/fl^ [vehicle (Veh), *n* = 5; T0901317 (T09), *n* = 10] and HMGB1^ΔHep^ (Veh, *n* = 3; T09, *n* = 7) mice were treated with either vehicle (5% carboxymethylcellulose) or LXR synthetic agonist T0901317 (oral gavage, 30 mg/kg per day) for four consecutive days. After 6-hour starvation on the last day, mice were sacrificed. (A) Liver tissue was then subjected to RT-qPCR analysis of the indicated LXR-dependent genes. (B) HMGB1^fl/fl^ (*n* = 10) and HMGB1^ΔHep^ (*n* = 12) mice were infected with either adenovirus expressing an LXR shRNA or a scramble (SCR) sequence and then subjected 7 days later to an F/R challenge. Liver steatosis was determined by Oil Red O (ORO) staining on liver sections, with the quantitative representation displayed on the right. (**C**) HMGB1^fl/fl^ [Veh, *n* = 4 to 6; rosiglitazone (Rosi), *n* = 7] and HMGB1^ΔHep^ (Veh, *n* = 4 to 6; Rosi, *n* = 8) mice were treated with either vehicle [5% dimethyl sulfoxide (DMSO) in saline] or PPARγ synthetic agonist rosiglitazone (30 mg/kg per day, intravenously) and were sacrificed 18 hours later. Liver tissue was then subjected to RT-qPCR analysis of the indicated PPARγ-dependent genes. (**D**) HMGB1^fl/fl^ (ShSCR, *n* = 7; ShPPARγ, *n* = 8) and HMGB1^ΔHep^ (ShSCR, *n* = 8; ShPPARγ, *n* = 7) mice were infected with either adenovirus expressing a PPARγ shRNA or a scramble (SCR) sequence and then subjected 7 days later to an F/R challenge. Liver steatosis was determined by Oil Red O staining on liver sections. Data are means ± SEM. *P < 0.05, **P < 0.01, ***P < 0.001, and ****P < 0.0001, HMGB1^fl/fl^ and HMGB1^ΔHep^ comparison, by unpaired Mann-Whitney comparison. ^$^*P* < 0.05 and ^$$^*P* < 0.01, for treatment effect by one-way ANOVA.

As LXRα and PPARγ are key lipogenic TFs involved in cholesterol metabolism and liver lipogenesis, the potential derepression of their activity induced by HMGB1 deletion could translate into liver steatosis. In this purpose, we examined the functional interdependence between HMGB1 and LXRα/PPARγ using pharmacological activation and adenoviral-mediated inhibition of LXRα/PPARγ in HMGB1^fl/fl^ and HMGB1^ΔHep^ mice (Fig. 5). First, to establish a possible causal link between the absence of HMGB1 and LXRα activity, the HMGB1^fl/fl^ and HMGB1^ΔHep^ mice were treated with a synthetic LXR agonist (T0901317) for four consecutive days (30 mg/kg, orally) (Fig. 5A and fig. S5A). Already before treatment, several LXRα-dependent genes (*Srebf1*, *Fasn*, *Elovl-6*, *Abcg5*, and *Abcg-8*) were up-regulated in the HMGB1^ΔHep^ livers (Fig. 5A). T0901317 treatment of HMGB1^fl/fl^ mice potently induced expression of LXR-dependent genes (*Srebf1*, *Fasn*, *Elovl-6*, *Scd-1*, *Abcg5*, and *Abcg-8*) in the liver compared to vehicle-treated HMGB1^fl/fl^ mice. HMGB1^ΔHep^ livers displayed a significantly higher response to T0901317 than HMGB1^fl/fl^ mice, with an enhanced expression of *Fasn*, *Elovl-6*, and *Abcg-5* (Fig. 5A). This higher response was corroborated by histological examination showing an increased Oil Red O staining in *Hmgb1*-deleted livers in mice subjected to the T0901317 treatment (fig. S5A). Together, these results indicate that the higher lipogenesis in HMGB1^ΔHep^ livers is likely due to an enhanced LXRα activity. To complement this study, and firmly establish the role of LXRα in the enhanced hepatic steatosis seen in HMGB1^ΔHep^ mice, LXRα expression was knocked down in vivo using an adenovirus expressing short hairpin RNA (shRNA) targeting the receptor (Ad-Sh*Lxr*α) (Fig. 5B and fig. S5, B to D). Seven days after viral infection, the hepatic LXRα, but not LXRβ, mRNA levels were reduced, showing that the expression of LXRα, along LXRα-dependent genes, was successfully blunted in Ad-Sh*Lxr*α injected animals compared to control animals injected with an adenovirus expressing a scrambled shRNA (Ad-Sh*SCR*), highlighting the efficacy of LXRα targeting (fig. S5, B and D). Consistent with the results presented above, Ad-sh*SCR*–treated HMGB1^ΔHep^ mice displayed increased hepatic steatosis compared to Ad-sh*SCR*–injected HMGB1^fl/fl^ mice either upon F/R (Fig. 5B and fig. S5B) or HFD feeding (fig. S5C), as shown by Oil Red O staining. However, knocking down LXRα (Ad-Sh*Lxr*α) induces a reduction of liver steatosis in HMGB1^ΔHep^ mice, suggesting that LXRα plays a role in the enhanced hepatic lipid synthesis of HMGB1^ΔHep^ mice.

Next, using a similar approach, we tested the potential contribution to steatosis of PPARγ (Fig. 5, C and D, and fig. S6). First, we activated PPARγ in HMGB1^fl/fl^ and HMGB1^ΔHep^ mice with a single dose of the PPARγ agonist rosiglitazone (25 mg/kg, intraperitoneally). Eighteen hours after the injection, *Hmgb1*-null livers showed a higher expression, compared to floxed livers, of PPARγ-responsive genes (*Lpl*, *Cd36*, and *Fabp4*) (Fig. 5C). To confirm the effect of PPARγ, we blunted its activity by injecting an adenovirus carrying an shRNA targeting PPARγ (Ad-sh*Ppar*γ) into HMGB1^fl/fl^ and HMGB1^ΔHep^ mice exposed to F/R (Fig. 5D) or HFD feeding (fig. S6B). After verifying the successful reduction of PPARγ levels (fig. S6A), we also observed that knocking down PPARγ (Ad-Sh*PPAR*γ) induces a strong reduction of liver steatosis in HMGB1^ΔHep^ mice compared to wild-type littermates (Fig. 5D and fig. S6B). Of note, blunting PPARγ expression did not modify the lipogenic response induced by HFD or F/R, between sh*SCR*- and sh*PPAR*γ-HMGB1^fl/fl^ mice (Fig. 5D and fig. S6B), undermining PPARγ contribution during liver steatosis. To avoid any wrong interpretation due to our genetic background, we subjected mice carrying a hepatocyte-specific *Ppar*γ deletion to an F/R challenge, and the results showed once again that there is no major contribution of hepatocyte PPARγ in the progression of F/R-induced liver steatosis (fig. S7). Together, it means that PPARγ per se is not a determinant trigger of hepatic lipogenesis, but *Hmgb1* deletion likely helps to remove the brake on PPARγ activity. These results suggest that the LXRα activity is responsible, together with PPARγ, for the enhanced hepatic lipid synthesis in *Hmgb1*-null livers. Together, all these findings support a repressive role of HMGB1 on hepatic lipogenesis through repression of LXRα and PPARγ activity.

### HMGB1 binds to LXRα and PPARγ target genes involved in lipogenesis

Having identified LXRα and PPARγ as potential targets for repression by HMGB1, we determined the molecular mechanisms by which HMGB1 is exerting this action. Considering the impact HMGB1 may have on chromatin compaction (*27*), we first performed an assay for transposase-accessible chromatin using high-throughput sequencing (ATAC-seq) to evaluate the global chromatin dynamics in the absence of hepatic HMGB1. Hepatocyte nuclei were purified from liver samples harvested from HMGB1^fl/fl^ and HMGB1^ΔHep^ mice upon CD feeding or after FR (fig. S8). At the basal state, the principal components analysis (PCA) analysis of the ATAC-seq peaks revealed no distinct pattern in chromatin states between both genotypes (fig. S8A), in reads alignment in a genome browser (fig. S8B), or in the open chromatin region (OCR) locations around transcription start sites (TSSs) (fig. S8C). In sharp contrast, F/R in HMGB1^fl/fl^ mice triggered significant changes in chromatin state compared to the CD condition (respectively 68,776 versus 47,725 OCRs), but similar modifications were detected in the liver chromatin from F/R HMGB1^ΔHep^ mice. Only four OCRs were differentially nucleosome-depleted between both genotypes, supported by the very high number of common aligned peaks (fig. S8D). PCA analysis, examination of TSS charts, and annotation pie chart confirmed the high similarity in the chromatin state of both libraries (fig. S8, E to G). A close visualization of aligned peaks in loci of lipogenic genes regulated by LXRα/PPARγ (*Srebf1*, *Scd-1*, *Cidec*, or *Fasn*) (fig. S8H) showed as expected the same chromatin state pattern between both genotypes. As presumed from this very low number of sites differentially opened in the chromatin between control and *Hmgb1*-null livers, enrichment analysis could not identify any statistically significant biological functions related to these modifications. Overall, the analysis of ATAC-seq datasets ruled out a putative model where HMGB1 may regulate hepatic lipid metabolism through chromatin packaging.

**Fig. 6.**
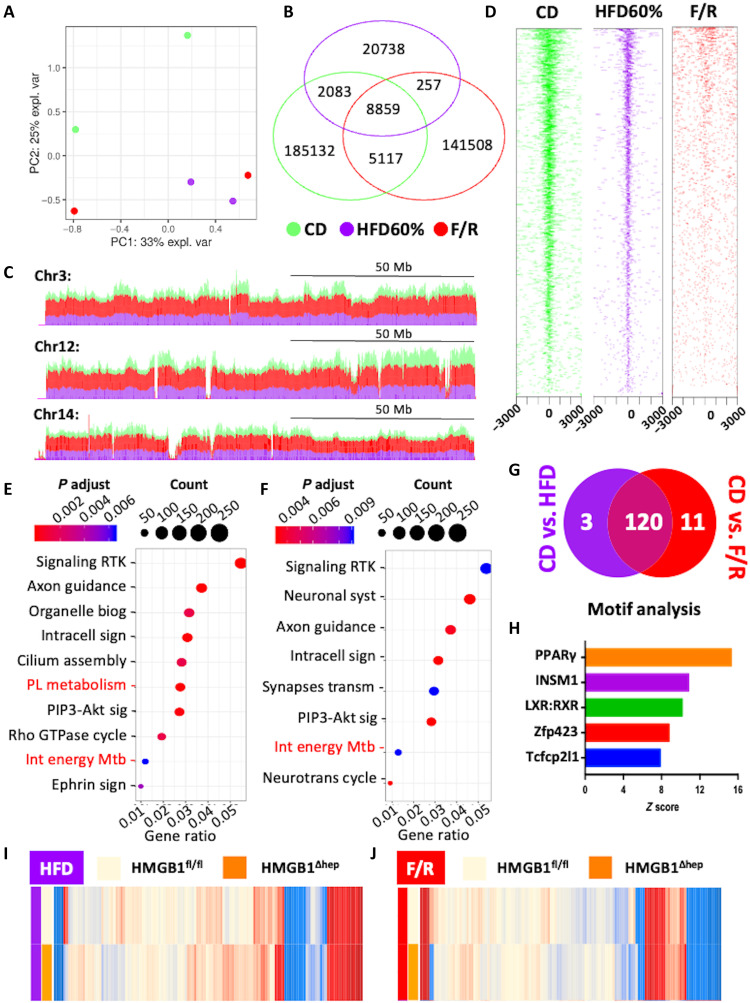
ChIP-seq identified a subset of LXR-responsive genes to be negatively regulated by HMGB1 during liver steatosis. (**A**) PCA score plot of ChIP-seq data of liver tissue from HMGB1^fl/fl^ mice on CD (green) or subjected to F/R (red) or HFD (purple). (**B**) Venn diagram showing the number of HMGB1 binding peaks, (**C**) UCSC genome browser of tracks (stacked) showing HMGB1 differential chromatin occupancy, and (**D**) average signal density profiles around transcription starting site in different nutritional states: CD (green) or during HFD (purple) or after F/R (red). (**E** and **F**) Functional enrichment analyses showing GO terms associated with the differential HMGB1 chromatin binding sites between (E) CD and HFD and (F) CD and F/R. (**G**) Venn diagram displaying shared enriched genes (*n* = 134) displaying a very high occupancy rate during fed state belonging to “Integration of energy metabolism” and “Phospholipid metabolism” GO functions compared to HFD (purple) and F/R (red). (**H**) Bar graph displaying consensus motifs in promoters of the 134 genes differentially occupied by HMGB1 via OPOSUM analysis; the bars represent the *z* score. (**I** and **J**) Heatmaps displaying the mean microarray expression levels for the 134 genes identified by ChIP-seq in liver from HMGB1^fl/fl^ (*n* = 4) and HMGB1^ΔHep^ (*n* = 4) mice subjected to either HFD (I) or F/R (J).

Next, we sought to determine, using chromatin immunoprecipitation combined with high-throughput sequencing (ChIP-seq), whether HMGB1 might exert its activity on gene transcription directly through its abilities to bind DNA. We first set up a reliable and robust ChIP protocol on cells in vitro, as HMGB1 ChIPing might be challenging (fig. S9, A to C) (*31*). Then, using frozen liver samples, we examined HMGB1 binding genome-wide in HMGB1^fl/fl^ under CD, under HFD, and after F/R (Fig. 6 and figs. S9 and S10). Of note, HMGB1 ChIP-seq was also performed on HMGB1^ΔHep^ livers, and these datasets were used as negative control to determine nonspecific signals. These background peaks were subtracted in libraries from HMGB1^fl/fl^ livers (fig. S9, D to F). Under CD feeding condition, 201,250 peaks were detected on the whole genome that were predominantly located in promoters (18.5%), introns (29.3%), and intergenic regions (32.6%) (fig. S10A). Only 155,854 and 32,006 peaks were detected under the F/R or HFD conditions, respectively, suggesting a significant remodeling of the HMGB1 binding pattern during metabolic stress, although the qualitative binding remains nearly the same (fig. S10A). The PCA plot of Fig. 6A demonstrates significant global differences in HMGB1 DNA occupancy between CD versus F/R and HFD. The Venn diagram confirmed this trend, with only a few peaks (8859) detected in common in the three conditions (Fig. 6B). The genome browser view of chromosomes 3, 12, and 14 exemplified the repositioning of HMGB1 upon nutritional stress (Fig. 6C). Along the same lines of observation, partitioning of HMGB1-bound sites by distance to TSSs confirmed the change in DNA occupancy of HMGB1. The results suggested that most HMGB1 sites located around the TSSs [±3000 base pairs (bp)] under CD feeding were not used under the F/R or HFD conditions (Fig. 6D). Enrichment analysis based on peaks differentially called in CD versus HFD feeding (Fig. 6E) and CD versus F/R (Fig. 6F) revealed that among several biological functions (GO categories), two are related to lipid metabolism as the “integration of energy metabolism” and “phospholipid metabolism” (Fig. 6, E and F). In these two GO categories, 134 genes displayed a very high occupation rate upon CD compared to F/R and HFD, and nearly 90% of these genes displayed a lower occupancy of HMGB1 in both challenges when compared to CD. These results suggest a common mechanism of regulation in F/R and HFD (Fig. 6G; full list in tables S1 and S2). To gain insight into the gene expression program regulated by HMGB1, we performed a motif identification analysis on 134 genes unveiled by the enrichment analysis. The oPOSSUM-3 motif tool revealed the binding motifs of the TFs of PPARγ and LXR, identifying this nuclear receptor among the top regulators (Fig. 6H). To functionally test whether the HMGB1 occupancy rate would have an incidence on the level of gene expression, we went back to the microarray data to measure the expression of the 134 genes identified in the enrichment analysis performed above. Of the 134 genes, 70 and 78 are up-regulated in HFD and F/R, respectively, in livers from HMGB1^ΔHep^ mice compared to HMGB1^fl/fl^ mice (Fig. 6, I and J), providing evidence for a negative correlation between the HMGB1 DNA occupation and the expression of metabolic-related genes identified in the ChIP-seq. These data demonstrate that HMGB1 may play a suppressive action on LXRα and PPARγ activities and, consequently, on the level of expression of their target genes.

Together, our data are in support of a model whereby at the basal state (CD), HMGB1 binds to chromatin loci to modulate the transcription of a number of genes controlled by LXRα and PPARγ, which are particularly involved in energy metabolism and lipogenesis.

### In vitro, HMGB1 exerts a repressive action on LXRα, but not on PPARγ

**Fig. 7.**
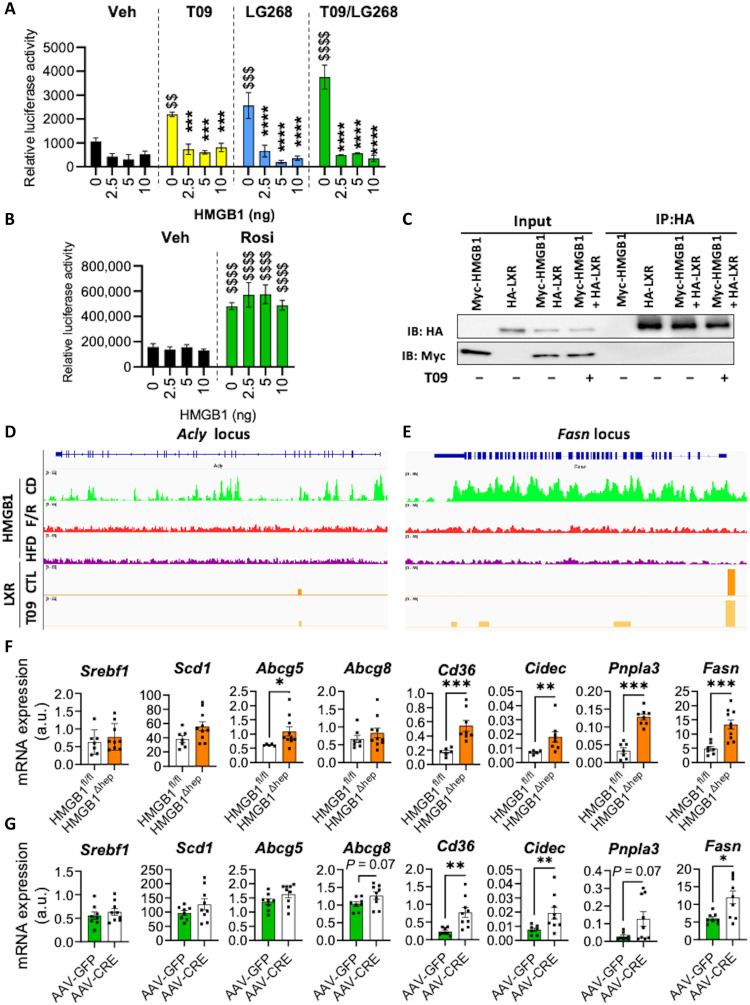
HMGB1 represses LXRα, but not PPARγ, transcriptional activity in vitro. (**A**) Effect of HMGB1 on LXRE-luciferase reporter activity. Ad293 cells were treated with DMSO (vehicle), T0901317 (T09) (0.1 μM), and/or LG286 (1 nM) for 14 hours. (**B**) Effect of HMGB1 on PPRE (PPAR responsive element)-luciferase reporter activity. Ad293 cells were treated with DMSO (vehicle) and rosiglitazone (Rosi; 1 μM, overnight). (**C**) Co-IP assay was performed to detect a potential interaction between HMGB1 and LXR in Ad293-transfected cells treated with DMSO (vehicle) or T0901317 (0.1 nM for 14 hours). Data are representative of three independent experiments. (**D** and **E**) IGV (Integrative Genomics Viewer) genome browser shot of HMGB1 and LXRα ChIP-seq data along the locus of *Acly* (D) and *Fasn* (E) gene loci. HMGB1 tracks in liver from HMGB1^fl/fl^ upon CD (green), upon HFD (purple), and after F/R (red). LXRα tracks from basal liver (dark orange) and T0901317 challenged liver (light orange). Gene loci displayed in gene model (blue) are displayed on the bottom track. (**F**) Gene expression of direct (*Srebf1*, *Scd-1*, *Abcg-5*, and *Abcg-8*) and indirect (*Cd-36*, *Cidec*, *Pnpla3*, and *Fasn*) targets of LXRα in livers of HMGB1^fl/fl^ (*n* = 7) and HMGB1^ΔHep^ (*n* = 9) mice. (**G**) Adult HMGB1^fl/fl^ mice were infected with either AAV8-TBG-GFP (*n* = 8) or AAV8-TBG-Cre (*n* = 9) to selectively generate *Hmgb1* deletion in hepatocytes in vivo, and expression of direct (*Srebf1*, *Scd-1*, *Abcg-5*, and *Abcg-8*) and indirect (*Cd-36*, *Cidec*, *Pnpla3*, and *Fasn*) responsive genes was determined using RT-qPCR. Data are means ± SEM of three independent experiments. *P < 0.05, **P < 0.01, ***P < 0.001, and ****P < 0.0001 by unpaired Mann-Whitney comparison or two-way ANOVA. ^$^*P* < 0.05, ^$$^*P* < 0.01, and ^$$$^*P* < 0.001, for treatment effect by two-way ANOVA.

Because HMGB1 is known to modulate chromatin structure and, therefore, regulate TF activity, we examined whether HMGB1 would inhibit LXRα- and PPARγ-dependent transcriptional activation in cell cultures transfected with luciferase reporter genes harboring response elements for these two receptors. Expression of HMGB1 markedly decreased LXR transcriptional activity already not only at the basal state but also after pharmacological activation by synthetic LXR agonists (T093911 or LG286) (Fig. 7A). On the contrary, HMGB1 did not repress the PPARγ activity either at the basal state or after rosiglitazone treatment (Fig. 7B), suggesting a specificity of inhibition toward LXRα. Next, we tested whether HMGB1 directly interacts with LXRα by in vitro coimmunoprecipitation (co-IP) assays. There was no interaction detected between a flagged (Myc)-HMGB1 and (HA)-LXRα (Fig. 7C). To investigate on a larger scale whether HMGB1 exerts repression on hepatic lipid metabolism through physical interactions with unidentified partner, we performed an interactome study and recorded protein partners pulled down after a co-IP assay combined with mass spectrometry (MS) proteomics from liver nuclear extracts in HMGB1^fl/fl^ and HMGB1^ΔHep^ mice upon HFD feeding or after FR (fig. S10, B and C). Twelve partners have been found to be differentially enriched between both genotypes, but none of them has already been related to liver lipogenesis (fig. S10, B and C). This is not only weakening a possible direct interaction with TFs involved in energy metabolism like LXRα or PPARγ but also compromising a model where HMGB1 may play an anchoring role on chromatin for repressor complexes (fig. S10, B and C). Therefore, we tested whether HMGB1-mediated inhibition of LXRα activity may occur by suppressing LXRα interaction with the DNA-encoding LXR target genes. The ChIP-seq data suggested that the localization of HMGB1 at specific gene loci correlated with its repressive role of LXR target genes such as *Acly* or *Fasn*. These two loci were significantly enriched in CD (green tracks) compared to HFD (purple tracks) and F/R (red tracks) (Fig. 7, D and E), and HMGB1 bound across the whole loci (Fig. 7, D and E) including promoters. In addition, these two HMGB1 repressed genes, *Acly* or *Fasn*, displayed a heterogeneous HMGB1 occupation pattern (fig. S10, E and F), with *Acly* promoter displaying a high occupation rate in the TSS as opposed to *Fasn* promoter (fig. S10, D and E). Of note, LXRα ChIP tracks (basal or T0901317-activated) for these two loci show a strong occupation of LXRα in DNA region also occupied by HMGB1 (Fig. 7, D and E, and fig. S10, D and E). This suggests that HMGB1 is not exerting its repressive effect only through TSS occupation. To further explore how HMGB1 could mediate its repressive action, motif analysis research was conducted on aligned HMGB1 peaks found ±3 kb flanking the TSS of 134 genes identified in the ChIP enrichment analysis. Using the MEME Suite, seven singular de novo motifs have been identified (fig. S10F), which are rather long (nearly 30 bp) and GC rich. The cross-identification with existing motifs using TomTom analysis tool shows some parenting with motifs bound by TF such as SP or KLF subfamily, rendering unlikely the possibility for HMGB1 to interfere and compete with LXRα and/or PPARγ binding sites (fig. S10F). Overall, these findings support the idea that HMGB1 is repressing LXRα and PPARγ transcriptional activities, not through a physical interaction with the receptor but rather through an unspecific and still ill-defined DNA occupation at the target genes of the receptors. This prompted us to extend the analysis to a series of key genes involved in lipogenesis by performing RT-qPCR experiments on liver samples from adult HMGB1^fl/fl^ and HMGB1^ΔHep^ mice fed CD, a condition under which HMGB1 repression was strong. The results showed a consistent up-regulation in the expression level of key lipogenic genes when HMGB1 was lacking in livers of HMGB1^ΔHep^ mice. The expression of direct or indirect LXRα/PPARγ target genes such as *Cd36*, *Cidec*, *Pnpla3*, or *Fasn* (Fig. 7F) was increased in the liver of these mice compared to their floxed littermates. To establish a causal link between the nuclear presence of HMGB1 and the mRNA expression level of the abovementioned genes, we deleted HMGB1 selectively in hepatocytes using the hepatocyte-specific promoter of the thyroxine-binding globulin (TBG) gene to express the Cre recombinase via an AAV8 vector (AAV8-TBG-Cre) in adult HMGB1^fl/fl^ mice. This strategy was validated by the lower levels of HMGB1 mRNA and protein levels detected in the liver of AAV8-TBG-Cre–expressing mice compared to the control group (fig. S11, A and B). Seven days after viral infection with the recombinant virus, the reduced *Hmgb1* expression resulted in up-regulation of LXRα-responsive genes, similarly to what is seen in liver of mice with a constitutive *Hmgb1* deletion in hepatocytes (Fig. 7G). This result supports a causal and repressive role for HMGB1 on the level of expression of this subset of genes. Overall, these findings support a model where HMGB1 is repressing directly LXRα and indirectly PPARγ transcriptional activities, which is not mediated by a direct physical interaction with the nuclear receptors but rather through a complex DNA occupation.

## DISCUSSION

Lipogenesis is a fundamental function of the liver to regulate and buffer the amount of circulating lipids, which could present a risk of cellular toxicity in the long run, for numerous tissues (*36*). Hepatic lipogenesis is therefore tightly regulated by a large number of factors, including TFs and nuclear proteins that together manage positive and repressive actions on gene transcription. These regulatory processes and their interplay are complex and only partly understood and have high relevance because of the high worldwide prevalence of NAFLD (*1*). Here, we unraveled a new mechanism regulating liver lipogenesis involving the nuclear factor HMGB1. Using both constitutive and induced knockouts of *Hmgb1* gene selectively in hepatocytes, we demonstrated that HMGB1, acting in the nucleus, exerts a potent repressive effect on LXRα and PPARγ activities and hepatic lipogenesis during metabolic stresses, such as F/R or HFD feeding, suggesting a protective role on the development of NAFLD.

The nuclear role of HMGB1 might be more complex than initially envisioned and may depend on cell type, nature of environmental signals, and the pathophysiological context. In the context of metabolic stress, we demonstrate in vitro, using primary culture of hepatocyte, that HMGB1 exerts its repressive effect on lipid metabolism in a cell-autonomous manner, thus supporting a model where HMGB1 remains inside the hepatocyte. One can presume that HMGB1 stays in the nucleus and/or translocates in the cytoplasm. Our ChIP-seq data showed that upon the nutritional challenges we have applied, HMGB1 leaves the chromatin, exemplified by reduced binding affinity to DNA and loss of TSS occupancy, triggering a number of changes in gene transcription. Other studies have described a similar impairment of DNA affinity by HMGB1 in cells subjected to stress (*31*, *37*). In a recent study, it was shown that in senescent cells, HMGB1 leaves the nucleus, leading to a significant change in gene expression (mostly up-regulation) and in chromatin topology (*31*), which is in agreement with our results in hepatocytes. Despite being poorly documented, it has also been described that HMGB1 in the nucleus may be both bound and unbound to DNA, and that even when unbound it may still reside within the nucleus during the cell cycle (*37*). This supports a model where upon stressors and/or outside signals, HMGB1 may dissociate from DNA but stays in the nucleus. Yet, the precise mechanisms regulating this biological event and the role of unbound HMGB1 within the nucleus remain unknown, and further experiments are required to understand the underlying mechanism.

Our data suggest that in response to microenvironmental signals, HMGB1 may dissociate from the chromatin, thus affecting biological functions, including metabolic processes. On CD, we found HMGB1 occupying 134 gene loci belonging to metabolic functions, which have been identified as depending on the activity of LXRα/ PPARγ. As LXRα is a key lipogenic TF involved in cholesterol metabolism and liver lipogenesis, the derepression of its activity induced by HMGB1 deletion logically translates into liver steatosis (*38*, *39*). It is less clear whether PPARγ is a significant trigger of liver steatosis in HMGB1^ΔHep^ mice. Our findings support the idea that there is no obvious contribution of hepatocyte PPARγ in the progression of HFD or F/R-induced liver steatosis (Fig. 5D and fig. S6). These findings not only suggest that PPARγ is, as opposed to LXRα, an unquestionable actor of liver lipogenesis but also indicate that the deletion of *Hmgb1* gene makes PPARγ a significant contributor of hepatosteatosis progression. In addition, in vitro assays using a luciferase reporter gene suggested that HMGB1 does not block or inhibit PPARγ directly, while the repression on LXRα is clear in the presence of agonist-mediated activation, supporting the notion that HMGB1 repression action is likely different between LXRα and PPARγ. It is therefore possible that DNA occupation may not be the only mechanism by which HMGB1 represses the activity of LXRα and PPARγ and another repressive scenario might be in play. A possibility is that PPARγ activity is indirectly increased by LXRα-enhanced activity. It is thus plausible but still hypothetical at this stage that the absence of HMGB1 leads to a higher LXRα activity translating in a higher SREBP1c activity known to produce endogenous ligands for PPARγ, as it has been demonstrated for adipose tissue (*40*). This hypothetical mechanism would mean that the increased PPARγ activity in the absence of HMGB1 is not mediated directly but is potentially under the dependence of LXRα.

Our ATAC-seq data helped to demonstrate that chromatin compaction was not regulated by HMGB1 under CD and during the nutritional challenges (fig. S8), suggesting that the HMGB1-mediated repression was likely not mediated through a nucleosomal reorganization. This hypothesis was important to test, as several reports demonstrated a key role of HMGB1 in the nucleosome arrangement remodeling associated to transcription modulation in vitro (*27*). At least in the in vivo context of liver steatosis, our results support a minor role for HMGB1 in regulating nucleosomal landscapes, which represents a significant layer of epigenetic control of transcription. However, our ChIP-seq data suggested DNA occupancy as a likely mechanism of repression. HMGB1 has a very high level of DNA occupation in the basal state and that it is located equally in the promoter region, CDS (coding sequence), and distal intergenic region. However, upon metabolic stress, HMGB1 appears to leave the chromatin, particularly the TSS regions (Fig. 6D). This suggests that HMGB1 DNA occupancy is correlated with changes in gene transcription, but the occupancy rate in the TSS is not necessarily related to the level of repression, as shown by two equally repressed genes (*Acly* and *Fasn*) with heterogeneous TSS occupation (fig. S10, E and F). Hence, occupancy appears to be an important factor but likely not the only one. Of note, our data using inducible *Hmgb1* deletion via AAV8-TBG-Cre show that the absence of HMGB1 consistently leads to the up-regulation of genes involved in hepatic lipogenesis, suggesting a causal relationship between HMGB1 and gene expression (Fig. 7, F and G). These results are corroborated by a study of Sofiadis *et al.* (*31*), depicting a map of HMGB1 binding genome-wide in senescent cells using a combination of RNA sequencing (RNA-seq), ChIP-seq, and Hi-C (chromatin conformation capture). In primary cells at the senescent state, HMGB1 leaves the chromatin, triggering profound changes in chromatin dynamics and gene transcription, in a similar fashion as seen by us. In addition, Hi-C data demonstrated that HMGB1 binds to TAD (topology-associated domain) boundaries, known to regulate chromatin topology and consequently gene expression. In addition to this paper, a recent study has also evoked an RNA-binding property as another functional layer for HMGB1 to regulate gene expression (*31*, *41*). Therefore, three-dimensional conformation and RNA binding represent additional mechanisms by which HMGB1 could mediate its repressive effect on LXRα, which will therefore be worthwhile to further investigate in the context of liver steatosis.

Overall, our study helped to uncover HMGB1-mediated repression of LXRα and PPARγ activities as a new mechanism modulating liver lipogenesis during metabolic stress. Boosting these functions of HMGB1 may constitute a new therapeutic approach to counteract the deleterious effect of enhanced LXRα/PPARγ activities in patients with NAFLD.

## MATERIALS AND METHODS

### Experimental design

This study aimed to decipher the precise role of the nuclear factor HMGB1 in hepatocytes during metabolic stress. For this, a cell-specific knockout mice model where *Hmgb1* gene is deleted specifically in hepatocytes (HMGB1^ΔHep^) and its control counterpart (HMGB1^fl/fl^), and mice model overexpressing *Hmgb1* (AAV8-CMV-HMGB1) and its control (AAV8-CMV-GFP) were subjected to nutritional stressors such as HFD and fasting/refeeding. A combination of OMICS studies has been used to nail down the potential mechanism behind HMGB1-repressive effect on hepatic lipogenesis such as microarray, ATAC-seq, or ChIP-seq. All studies identified lipid metabolism as a crucial function and TFs LXRα and PPARγ as key pieces that might be repressed by HMGB1. In vivo studies using adenovirus-mediated shRNA expression targeting LXRα/PPARγ were used to functionally test the interdependence of HMGB1 and LXRα/PPARγ. In vitro assays were used to measure how HMGB1 could regulate the transcriptional activation using specific responsive elements (REs) containing luciferase reporter. For in vivo studies, adult age-matched Cre^+/−^ carrying *Hmgb1* floxed gene called HMGB1^ΔHep^ mice and their control Cre^−/−^ carrying *Hmgb1* floxed gene named HMGB1^fl/fl^ littermates were co-housed to reduce variability. Animal numbers for each study type were determined by the investigators on the basis of data from previous similar experiments or from pilot studies. For OMICS studies, displayed animals were chosen as representative from the whole cohort as follows: (i) Four animals per genotype per challenge for microarray, (ii) two animals per genotype per challenge for ChIP-seq, and (iii) two animals per genotype per challenge for ATAC-seq have been analyzed. For neutral lipid analysis and histology experiments, sample identities were not known in most cases and were randomized. For in vitro studies, at least three biological replicates were used in three separate experiments.

### Mouse phenotyping

Breeding and experimental procedures were performed in accordance with institutional guidelines for animal research and were approved by the Animal Care and Use Ethics Committee US006 CREFRE-CEEA-122 (protocol 17/1048/03/20). Animals were housed in temperature- and humidity-controlled facilities under a 12-hour light period with free access to food and water. All animals were aged between 2 and 3 months at the beginning of the experimentations. C57BL/6 mice were purchased from Envigo Laboratories (Gannat, France). Hepatocyte-specific deletion of *Hmgb1* gene noted HMGB1^ΔHep^ was generated by crossing Alb-CRE^+/−^ (The Jackson Laboratory, Bar Harbor, ME, USA) with *Hmgb1* floxed mice noted HMGB1^fl/fl^ (a gift from R. F. Schwabe, Columbia University, NY, USA), and littermates Alb-CRE^−/−^ HMGB1^Flox/Flox^ (HMGB1^fl/fl^) were used as control. Hepatocyte-specific deletion of *Ppar*γ gene noted PPARγ^ΔHep^ was generated by crossing Alb-CRE^+/−^ (The Jackson Laboratory, Bar Harbor, ME, USA) with *Ppar*γ floxed mice noted PPARγ^fl/fl^ (a gift from W. A. Wahli, University of Lausanne, Switzerland), and littermates Alb-CRE^−/−^ PPARγ^Flox/Flox^ (PPARγ^fl/fl^) were used as control. At the time of sacrifice, tissues and organs were dissected, weighted, directly snap-frozen in liquid nitrogen, and stored at −80°C.

### Genotyping

DNA extraction and PCR were performed using a Kapa mouse genotyping kit (Kapa Biosystems, Wilmington, MA, USA) according to the manufacturer’s protocol. PCRs were performed using the following primers: Alb-CRE,

5′-ACCGGTCGATCGAAACGAGTGATGAG-3′ (forward)

and 5′-AGTGCGTTCGAACGCTAGAGC-3′ (reverse); LoxP1,

5′-TAAGAGCTGGGTAAACTTTAGGTG-3′ (forward)

and 5′-GAAACAGACAAGCTTCAAACTGCT-3′ (reverse); LoxP2,

5′-TGACAGGATACCCAGTGTTAGGGG-3′ (forward) and 5′-CCAGAGTTTAATCCACAGAAGAAA-3′ (reverse).

### Interventional experiments

For diet-induced obesity experiments, mice were fed with a normal CD (Research Diets, New Brunswick, NJ, USA) or an HFD (HFD60%, Research Diets, New Brunswick, NJ, USA) for 12 or 24 weeks. For the fasting-refeeding, mice under normal CD were starved 6 hours from Zeitgeber 14 (ZT14) and refed for 8 hours with the CD and 20% glucose (Sigma-Aldrich, St. Louis, MO, USA) in the drinking water. Body composition was assessed using EchoMRI (Echo Medical Systems, Houston, TX, USA).

Indirect calorimetry was performed after 24 hours of acclimatization in individual cages. Oxygen consumption, carbon dioxide production, and food and water intake were measured (Phenomaster, TSE Systems, Bad Homburg v.d.H, Germany) in individual mice at 15-min intervals during a 24-hour period at constant temperature (22°C). The respiratory exchange ratio ([RER] = *V*co_2_/*V*o_2_) was measured.

For *Hmgb1* gene deletion at adult age, HMGB1^fl/fl^ male mice at 8 weeks of age were injected intravenously with 10^11^ genomic copies per mouse with AAV8 containing a liver-specific promoter and TBG promoter driving either GFP or Cre recombinase (Penn Vector Core, University of Pennsylvania, PA, USA) to generate control mice noted AAV-GFP or liver-specific HMGB1 knockout noted AAV-CRE. Seven days after injections, animals were euthanized.

To knock down LXR and PPARγ, adult male HMGB1^fl/fl^ and HMGB1^ΔHep^ mice (8 to 12 weeks old) were injected intravenously with an adenovirus expressing an shRNA targeting LXRα (provided by C. Postic, Cochin Institute, Paris, France) or PPARγ (provided by G. Panasyuk, Necker Institute, Paris, France). For both adenovirus protocols, 10^9^ adenoviral infectious particles were diluted in 0.9% NaCl and administered retro-orbitally in a total volume of 100 μl per animal. Seven to 10 days after injection, control (scramble RNA noted sh*SCR*) and sh*LXR*α- or sh*PPAR*γ-expressing mice were subjected to fasting/refeeding challenges as described previously. To study HFD-induced liver steatosis, mice were first subjected to 4-week HFD60%, then injected with sh*SCR*, sh*LXR*α, or sh*PPAR*γ, and euthanized 7 to 10 days after injections.

For HMGB1, overexpression was mediated using AAV. Control virus (AAV8-CMV-GFP) or AAV8-CMV-mHMGB1 (Vector Biosystems Inc., Malvern, PA, USA) was administered retro-orbitally. For both adenovirus, 10^12^ genomic copies were diluted in 0.9% NaCl and administered retro-orbitally in a total volume of 150 μl per mouse. Two days after administration, both groups were fed for 12 weeks with HFD60%.

For insulin acute injection, CD or HFD60% fed HMGB1^fl/fl^ and HMGB1^ΔHep^ mice were fasted for 4 hours and then injected intraperitoneally with human insulin (0.75 U/kg) and mice were sacrificed 15 min later.

For LXR in vivo activation, the synthetic agonist T0901317 (30 mg/kg; Bertin Bioreagent, Montigny le Bretonneux, France) was administered orally by four consecutive daily gavages on 8-week-old HMGB1^fl/fl^ and HMGB1^ΔHep^ adult male mice. Mice were starved 1 hour before the fourth gavage and maintained starved for five more hours before euthanasia.

For hepatic VLDL-triacylglycerol production assay, 8-week-old HMGB1^fl/fl^ and HMGB1^ΔHep^ adult male mice that fasted overnight received an intravenous injection of 10% tyloxapol (500 mg/kg) (Sigma-Aldrich, St. Louis, MO, USA, T8761). Blood was collected from the tail vein at 0, 1, 2, 3, and 4 hours for triglyceride assays.

### Glucose/insulin/pyruvate tolerance test

Glucose, insulin, and pyruvate tolerance tests were performed under CD or after 12 weeks of HFD after an overnight fast. Glucose (Sigma-Aldrich, St. Louis, MO, USA, G8270) was orally administered at 1.5 g/kg dose, insulin was injected intraperitoneally at 0.75 U/kg, and pyruvate (Sigma-Aldrich, St. Louis, MO, USA, P2256) was administered by intraperitoneal injection at 1.5 g/kg. For all tolerance tests, the glycemia evolution was then monitored at the tail vein using an Accu-Check glucometer (Roche). Plasma insulin (Mercodia, Uppsala, Sweden) was determined by enzyme-linked immunosorbent assay (ELISA) in the fasted state or at indicated times.

### Primary hepatocyte isolation

Mouse hepatocytes were isolated as previously described via two-step collagenase perfusion as described by Fortier *et al.* (*42*). Hepatocytes were allowed to attach for 90 min on collagen-coated plates in RPMI containing 10% fetal bovine serum (Gibco), followed by overnight starvation in serum-free medium before experiments (lipogenesis and β-oxidation assay).

### Lipogenesis assays

For in vitro measurement 1 day after isolation, primary hepatocytes were serum-starved for 3 hours and incubated for 3 hours with [1-^14^C]acetate (1 μCi/ml; PerkinElmer, Boston, MA) and 5.5 mM of nonlabeled (cold) glucose in Dulbecco’s modified Eagle’s medium (DMEM). At the end of incubation, cells were washed twice with 1× cold PBS and harvested into 0.25 ml of 0.1% SDS for subsequent protein measurement and total lipid extraction with 1 ml of chloroform/methanol (2:1, v/v). Lipid extracts were washed with 70% ethanol and then dissolved into chloroform/methanol (2:1, v/v). Radioactivity was measured on a multipurpose scintillation counter (LS 6500, Beckman Coulter). All assays were performed in duplicate, and data were normalized to cell protein content.

For in vivo measurement of lipogenesis activity, animals were fasted for 6 hours at ZT14 and received an intraperitoneal bolus of glucose (2 mg/g) containing [3-^3^H]d-glucose (0.4 μCi/g) (PerkinElmer, NET331C, Waltham, MA, USA). After 1 hour, liver, epididymal, subcutaneous, and brown adipose tissues were collected and snap-frozen in liquid nitrogen.

### Liver neutral lipid analysis

Hepatic lipids were extracted by the “Folch” procedure before being quantified using MS. Briefly, 50 mg of liver was homogenized in 1 ml of water:methanol (1:2, v/v) and 5 mM EGTA. Lipids were then extracted using a methanol:chloroform:water (2.5:2.5: 1.7, v/v) mix. After a solid-phase extraction, purification, and desiccation, all lipids were eluted in ethyl acetate and analyzed by gas chromatography combined with MS (GC-MS) (ISQ Thermo).

### Lipogenesis using ^2^H_2_O

For de novo lipogenesis measurement using ^2^H_2_O, mice were subjected to 4% ^2^H_2_O (Sigma-Aldrich, 151882) in the drinking water during 3 weeks. Mice were then euthanized, and plasma and liver tissue were collected and flash-frozen in liquid nitrogen. ^2^H_2_O content in body water of the mice was determined in plasma according to the protocol adapted from Daurio *et al.* (*43*) and was approximately 3%.

Total fatty acids (20 mg of liver) were extracted from Bligh and Dyer in CH_2_Cl_2_/MeOH 2% AA (acetic acid)/H_2_O (2.5:2.5:2, v/v/v) in the presence of the internal standard TG19 at 4 μg. The lipid extract was hydrolyzed in 1 ml of KOH (0.5 M in MeOH) at 55°C for 30 min. After the addition of 1.5 ml of MeOH, 2 ml of H_2_O, and 2.5 ml of CH_2_Cl_2_, total FA (formic acid) extract was dried and derivatized in pentafluorobenzyl esters in 1% PFB-Br (pentafluorobenzyl bromide) and 1% DIPEA (N,N-diisopropylethylamine) in ACN (acetonitrile) (50 μl) at room temperature for 20 min. Samples were dried and dissolved in EtOAc (20 μl) for the injection in GC-MS. The total labeled FA analysis was performed on a Thermo Fisher Scientific Trace GC system connected to a Thermo Fisher Scientific TSQ8000 EVO triple quadrupole detector using an HP-5MS capillary column (30 m by 0.25 mm, 0.25-μm film thickness). Oven temperature was programmed as follows: 180°C for 1 min, 8°C/min to 220°C, 2°C/min to 260°C, 10°C/min to 300°C, and then the temperature was kept constant for 2 min. The carrier gas was helium (1 ml/min). The injector, the transfer line, and the ion source temperature were at 270°C, 300°C, and 210°C, respectively. One microliter of sample was injected in splitless mode. TSQ8000 was operated in chemical ionization in negative mode (methane at 1 ml/min) in full scan. For data processing, GC-MS analysis produced a mass spectrum for each FA, which contains the relative abundance of each isotopologue, and their integrations gave the isotopic cluster. For each FA, the lightest (unlabeled) isotopologue is denoted M+0; e.g., PFB-palmitate M+0 has a mass of 255.3, whereas the isotopologue with 1 atom [^2^H]PFB-palmitate (M + 1) has a mass of 256.3. Isotopologue distributions were obtained from the corresponding isotopic clusters after correction for natural abundance of carbon and nontracer elements using the software IsoCor. Last, ^2^H enrichment, which represents the mean content in tracer atoms (^2^H) within the molecule, was calculated from the corresponding IDs as detailed by Millard *et al.* (*44*). De novo lipogenesis was calculated using the following formula: A00 × palmitate ^2^H enrichment/(body water ^2^H enrichment × *n*), where *n* is the number of exchangeable hydrogens, which is assumed to be 22 (*45*, *46*).

### Microarray gene expression studies

Gene expression profiles were performed at the GeT-TRiX facility (Géntoul, Génopole Toulouse Midi-Pyrénées) using Agilent Sureprint G3 Mouse GE v2 microarrays (8×60K, design 074809) following the manufacturer’s instructions. For each sample, cyanine-3 (Cy3)–labeled complementary RNA (cRNA) was prepared from 200 ng of total RNA using a One-Color Quick Amp Labeling kit (Agilent) according to the manufacturer’s instructions, followed by Agencourt RNAClean XP (Agencourt Bioscience Corporation, Beverly, MA). Dye incorporation and cRNA yield were checked using a DropSense 96 UV/VIS droplet reader (Trinean, Belgium). A total of 600 ng of Cy3-labeled cRNA was hybridized on the microarray slides following the manufacturer’s instructions. Immediately after washing, the slides were scanned on Agilent G2505C Microarray Scanner using Agilent Scan Control A.8.5.1 software, and fluorescence signal was extracted using Agilent Feature Extraction software v10.10.1.1 with default parameters.

### Microarray data statistical analysis

Microarray data were analyzed using R (*47*) and Bioconductor packages (*48*). Raw data (median signal intensity) were filtered, log_2_-transformed, and normalized using the quantile method (*49*) with the limma package (*50*).

A model was fit using the limma lmFit function (*50*). Pairwise comparisons between biological conditions were applied using specific contrasts. In cases where Agilent has multiple probe sequences for the same gene, the probe with the best *P* value was selected. Probes with a *P* value of ≤0.01 were considered to be differentially expressed between conditions.

Normalized log intensities were averaged (*n* = 4) within each group, and heatmaps were generated with the ComplexHeatmap package (*51*). Venn diagrams were generated with the Vennerable package (https://github.com/js229/Vennerable). Functional pathway enrichment was performed in R using the hypergea package’s hypergeometric test (https://cran.r-project.org/package=hypergea). GO annotations were obtained using biomaRt (*52*), and the graphite package (*53*) was used to obtain pathways from the Reactome database. ChEA (ChIP Enrichment Analysis) (*54*) was interrogated via the Enrichr website (*55*), and tabular results were imported into R. Bar charts were constructed using ggplot2 (*56*). The network of pathways largely shared between F/R and HFD was constructed in R as csv files that were imported into Cytoscape (*57*).

### Chromatin immunoprecipitation sequencing

Briefly, frozen liver biopsies (100 to 200 mg) harvested from HMGB1^fl/fl^ and HMGB1^ΔHep^ mice under CD, upon HFD60% or after F/R, were minced and fixed at room temperature in PBS–1% formaldehyde (Sigma-Aldrich, St. Louis, MO, USA, 47608) for 20 min. After sonication, ChIP was performed using anti-HMGB1 antibody (Abcam, ab18256, Cambridge, UK). Immunoprecipitated DNA was subjected to library preparation and single-end sequencing on NextSeq 500 at EMBL GeneCore (Heidelberg, Germany).

### Transposase-accessible chromatin using high-throughput sequencing

Flash-frozen liver biopsies were sent to Active Motif to perform the ATAC-seq assay. The tissue was manually dissociated, isolated nuclei were quantified using a hemocytometer, and 100,000 nuclei were tagmented as previously described (*58*), with some modifications based on (*59*) using the enzyme and buffer provided in the Nextera Library Prep Kit (Illumina). Tagmented DNA was then purified using a MinElute PCR purification kit (Qiagen), amplified with 10 cycles of PCR, and purified using Agencourt AMPure SPRI beads (Beckman Coulter). The resulting material was quantified using the Kapa Library Quantification Kit for Illumina platforms (Kapa Biosystems) and sequenced with PE42 sequencing on a NextSeq 500 sequencer (Illumina).

### ATAC-seq and ChIP-seq data analysis

ATAC-seq and ChIP-seq reads were first mapped to the mouse genome UCSC build hmm10 using Bowtie2 2.2.8 (*60*). Aligned reads were then filtered to keep only matched pairs and uniquely mapped reads. Peaks were called with MACS2 2.2.1 (*61*) algorithm using a mappable genome size of 2.73 × 10^9^. To process ChIP-seq datasets, MACS2 was run with the “Delta” genotype as a negative control, as in this condition the HMGB1 protein expression is reduced by 90% and signal detected in Delta libraries, defined as background noise, was subtracted from the “Flox” libraries. ATAC-seq datasets were processed without a control file and with the –nomodel option. Called peaks that were on the ENCODE blacklist of known false ChIP-seq peaks were removed. Signal maps and peak locations were used as input to the statistical analysis performed with the R package ChIPseeker (*62*). DESeq2 (*63*) was used to identify differential binding sites and differential open chromatin profiles. Motifs and GO enrichment analysis were respectively performed using JASPAR (*64*) and the R package ReactomePA (*65*). LXRα ChIP tracks have been extracted from the Gene Expression Omnibus database (GSE35262a) based on the published work from S. Mandrup’s laboratory (*66*).

### Histology

Tissue samples were fixed in 10% formalin (Sigma-Aldrich, St. Louis, MO, USA, HT501128) for 24 hours and then incubated at 4°C in 70% ethanol before being paraffin-embedded or in 30% sucrose before being cryo-embedded with Tissue-Tek O.C.T. (Sakura FineTek Europe, Alphen aan den Rijn, The Netherlands). Paraffin-embedded livers were sliced at 5 μm. For PAS reaction, sections were incubated in 0.5% periodic acid in water for 5 min and then transferred to Schiff reagent (Sigma-Aldrich, St. Louis, MO, USA, 3952016) for 15 min. Sections were counterstained with Mayer’s hematoxylin (Sigma-Aldrich, St. Louis, MO, USA, MHS16) before mounting. Liver cryosections were post-fixed with 10% formalin 15 min before staining with Oil Red O (Sigma-Aldrich, St. Louis, MO, USA, MHS16). HMGB1 immunohistochemistry was performed using rabbit anti-HMGB1 (1:1000; ab18256, Abcam, Cambridge, UK). After counterstaining with hematoxylin, slides were mounted with aqueous mounting medium. Stained slides were scanned using a Nanozoomer scanner (Hamamatsu Photonics, Hamamatsu City, Japan). Image quantification was performed using ImageJ freeware (National Institutes of Health, USA).

### Western blotting

Tissues were homogenized in radioimmunoprecipitation assay buffer (20 mM tris,150 mM NaCl, 1 mM EDTA, 1 mM EGTA, 1% Triton X-100, 2.5 mM tetrasodium pyrophosphate, 1 mM β-glycerophosphate, and 1 mM sodium orthovanadate) containing protease and phosphatase inhibitors (Sigma-Aldrich, St. Louis, MO, USA) using a Precellys sample lyzer (Bertin Technologies, Montigny le Bretonneux, France). Western blots were performed using standard procedures using antibodies against HMGB1 (1:1000, ab18256, Abcam, Cambridge, UK), phospho-AKT S473 (1:1000, CST 4060, Cell Signaling Technology, Danvers, MA, USA), total AKT (1:1000, CST 9272, Cell Signaling Technology, Danvers, MA, USA), hemagglutinin (HA) (1:1000, CST 3724 Cell Signaling Technology, Danvers, MA, USA), PPARγ (1:1000, C26H12, Cell Signaling Technology, Danvers, MA, USA), Myc-tag (1:1000, CST 2276, Cell Signaling Technology, Danvers, MA, USA), and glyceraldehyde-3-phosphate dehydrogenase (GAPDH) (1: 2000, ab181602, Abcam, Cambridge, UK), used as a loading control.

Detection of high–molecular weight proteins was performed using capillary electrophoresis (Wes, SimpleProtein, San Jose, CA, USA) using antibodies against FAS (CST 3189, Cell Signaling Technology, Danvers, MA, USA), ACC (CST 3662, Cell Signaling Technology, Danvers, MA, USA), and ACLY (ab40793, Abcam, Cambridge, UK).

### Reporter assay

For reporter assay, Ad293 cells were cultured in 96-well plates with DMEM containing 10% FB Essence (Avantor Seradigm, USA) and transfected using Transit-LT1 (Mirus Bio, Madison, WI, USA). For LXR activity, cells were transfected with plasmid encoding four LXR response elements fused with luciferase, Myc-HMGB1, human HA-LXR (HA-hLXR), and RXR. For PPARγ activity, cells were transfected with plasmid encoding four PPAR response elements fused with luciferase. Myc-HMGB1 plasmid was purchased from OriGene. Twenty-four hours after transfection, cell medium was changed to DMEM containing 2% charcoal-stripped and dialyzed medium with 0.1 μM T0901317 and/or 1 μM LG100268 (noted LG268) (Cayman Chemical, USA) for LXR or 1 μM rosiglitazone (Sigma-Aldrich, St. Louis, MO, USA, R2408) for PPARγ. After overnight treatment, luciferase activity was assayed using a luciferase assay system (Promega, USA). Bioluminescence was quantified using a luminometer and normalized to β-galactosidase activity.

### Coimmunoprecipitation

Ad293 cells were plated in a six-well plate and transfected as previously described with 1 μg of HA-hLXR and/or HMGB1 plasmids. Twenty-four hours after transfection, cells were treated with 0.1 μM T0901317 overnight and lysed in IP buffer [20 mM tris-HCl (pH 8), 100 mM NaCl, 0.1% NP-40, 10% glycerol, 2 μM phenylmethylsulfonyl fluoride, and 1 mM dithiothreitol (DTT)] supplemented with anti-protease and anti-phosphatase cocktails. IP was performed using HA-conjugated beads (Sigma-Aldrich) for 2 hours at 4°C, following wash step, beads were resuspended in 2× Laemmli buffer, and Western blot was performed as previously described.

Liver nuclear extracts from HMGB1^fl/fl^ (*n* = 3) and HMGB1^ΔHep^ (*n* = 3) mice were prepared out of a 50-mg frozen liver biopsies homogenized using a Dounce homogenizer in a two-step protocol as described previously (*67*). HMGB1 co-IP was performed overnight at 04°C on nuclear extract using anti-HMGB1 antibody (ab18256, Abcam, Cambridge, UK) and A/G Sepharose beads (GE Healthcare, Chicago, Illinois, IL, USA). After elution in 50 μl in Laemmli sample buffer, all samples were analyzed using MS-based quantitative proteomics.

### LC-MS/MS analysis for proteomics

Protein eluates were digested using S-Trap microspin columns according to the manufacturer’s protocol (DOI: 10.1021/acs.jproteome.8b00505), except that samples were reduced with 200 mM DTT (20 mM final) for 10 min at 95°C under agitation and alkylated with 1 M iodoacetamide (60 mM final) for 30 min at room temperature in the dark. The pooled eluates were dried down and resuspended in 17 μl of 2% ACN, 0.05% trifluoroacetic acid (TFA), vortexed, and sonicated for 10 min before injection. Samples were analyzed using an Ultimate 3000 nanoRS system coupled to a Q-Exactive Plus mass spectrometer (Thermo Fisher Scientific, Bremen, Germany). Five microliters of each sample was loaded onto a C18 precolumn (300 μm inner diameter by 5 mm) at 20 μl/min in 2% ACN and 0.05% TFA. After 5 min of desalting, the precolumn was switched online with the analytical C18 nanocolumn (75 μm inner diameter by 15 cm, packed in-house) equilibrated in 95% solvent A (5% ACN and 0.2% FA) and 5% solvent B (80% ACN and 0.2% FA). Peptides were eluted using a 5 to 25% gradient of solvent B for 80 min and then a 25 to 50% gradient of solvent B for 30 min at a flow rate of 300 nl/min. Q-Exactive Plus was operated in data-dependent acquisition mode. MS survey scans were acquired in the Orbitrap, on the 350 to 1500 mass/charge ratio (*m*/*z*) range, with the resolution set to a value of 70,000 at *m*/*z* 400. The 10 most intense multiply charged ions (up to 2+) were selected and fragmented by higher-energy collisional dissociation, and the resulting fragments were analyzed in the Orbitrap at 17,500 resolution. Dynamic exclusion was used within 30 s with a 10-ppm (parts per million) tolerance to prevent repetitive selection of the same peptide.

### Bioinfomatic analysis of LC-MS/MS data

Acquired MS and MS/MS data as raw MS files were converted to the mzDB format to generate peak lists (*68*). Peak lists were searched against UniProtKB/Swiss-Prot protein database with *Mus musculus* taxonomy (96,216 sequences) in Mascot search engine (version 2.6.2, Matrix Science, London, UK). Cysteine carbamidomethylation was set as a fixed modification. Methionine oxidation and acetylation of protein N terminus were set as variable modification. Up to two missed trypsin/P cleavages were allowed. Mass tolerances in MS and MS/MS were set to 10 ppm and 0.8 Da, respectively. Proline software (*69*) was used for protein validation at 1% false discovery rate, both at peptide and protein levels, and for label-free quantification of identified proteins. Missing values of extracted signals were independently replaced using Gaussian imputation. After log_2_ transformation of the data, the values of the technical replicates were averaged for each analyzed sample. To compare the two conditions, an unpaired two-tailed Student’s *t* test was performed. Proteins were considered significantly represented when their absolute log_2_-transformed fold change is superior or equal to 1 and their *P* value is under or equal to 0.05. Volcano plots were drawn to visualize significant protein abundance variations between the two compared conditions. They represent log_10_ (*P* value) according to the log_2_ ratio.

### Gene expression

RNA was extracted using a GenJET RNA purification kit (Thermo Fisher Scientific, Waltham, MA, USA) and deoxyribonuclease treatment (Qiagen, Hilden, Germany). After dosage with DropSense16 (Trinean, Gentbrugge, Belgium), reverse transcription was performed using the High-Capacity cDNA Reverse Transcription Kit (Applied Biosystems, Foster City, CA, USA) according to the manufacturer’s protocol. RT-qPCR was performed with indicated primer pairs; gene expression is normalized using *36b4* reference gene expression. Primer sequences are available in table S3.

### Microfluidic qPCR

Expression analyses of lipogenesis-related genes (table S3) were performed by qPCR with Fluidigm Biomark technology (Genome & Transcriptome GenoToul Platform). First-strand cDNA templates were preamplified with Preamp Master Mix (Fluidigm), and reactions were achieved in a Fluidigm Biomark BMK-M-96.96 plate according to the manufacturer’s recommendations. Relative gene expression values were determined using the 2^−∆∆*C*T^ method. The expression analysis data are an average of 7 individuals for HMGB1^fl/fl^ mice and 10 individuals for HMGB1^ΔHep^ mice. As described before, the *36B4* gene expression levels were used for data standardization.

### Plasma analysis

Whole blood is drawn out from the inferior vena cava after euthanasia, and plasma is prepared after centrifugation (5 min; 4°C; 8000 rpm). Circulating aspartate amino transferase and alanine amino transferase levels were determined in plasma by the Phénotypage-CREFRE facility using a Pentra400 biochemical analyzer (HORIBA Medical, Kyoto, Japan). HMGB1 circulating levels were assessed by ELISA (ST51011, IBL International, Hamburg, Germany) on 10 μl of plasma, according to the manufacturer guidelines.

### Statistics

Analyses were performed using GraphPad Prism 7 (GraphPad Software, La Jolla, CA, USA). Potential outliers were identified using ROUT algorithm (GraphPad Software) and removed from analysis. All data are expressed as means ± SEM, except otherwise indicated. Statistical significance was determined by Mann-Whitney, one-way analysis of variance (ANOVA), or two-way ANOVA, followed by a Sidàk post hoc test. *P* values of <0.05 were considered significant (*P < 0.05; **P < 0.01; ***P < 0.001; ****P < 0.0001).

## Notes

View/request a protocol for this paper from *Bio-protocol*.
