# Supplementary material for: Nuclear HMGB1 protects from nonalcoholic fatty liver disease through negative regulation of liver X receptor
Source: Sci Adv. 2022 Mar 25;8(12):eabg9055. doi: 10.1126/sciadv.abg9055 (PMC8956270; doi:10.1126/sciadv.abg9055)
Supplement: Supplementary file 1 — Figs. S1 to S11 Tables S1 to S3 [file sciadv.abg9055_sm.pdf]

Science Advances
AAAS

Supplementary Materials for

Nuclear HMGB1 protects from nonalcoholic fatty liver disease through negative regulation of liver X receptor

Jean Personnaz, Enzo Piccolo, Alizée Dortignac, Jason S. Iacovoni, Jérôme Mariette, Vincent Rocher, Arnaud Polizzi, Aurélie Batut, Simon Deleruyelle, Lucas Bourdens, Océane Delos, Lucie Combes-Soia, Romain Paccoud, Elsa Moreau, Frédéric Martins, Thomas Clouaire, Fadila Benhamed, Alexandra Montagner, Walter Wahli, Robert F. Schwabe, Armelle Yart, Isabelle Castan-Laurell, Justine Bertrand-Michel, Odile Burlet-Schiltz, Catherine Postic, Pierre-Damien Denechaud, Cédric Moro, Gaelle Legube, Chih-Hao Lee, Hervé Guillou, Philippe Valet, Cédric Dray, Jean-Philippe Pradère*

*Corresponding author. Email: jean-philippe.pradere@inserm.fr

Published 25 March 2022, *Sci. Adv.* **8**, eabg9055 (2022)
DOI: 10.1126/sciadv.abg9055

This PDF file includes:

Figs. S1 to S11
Tables S1 to S3

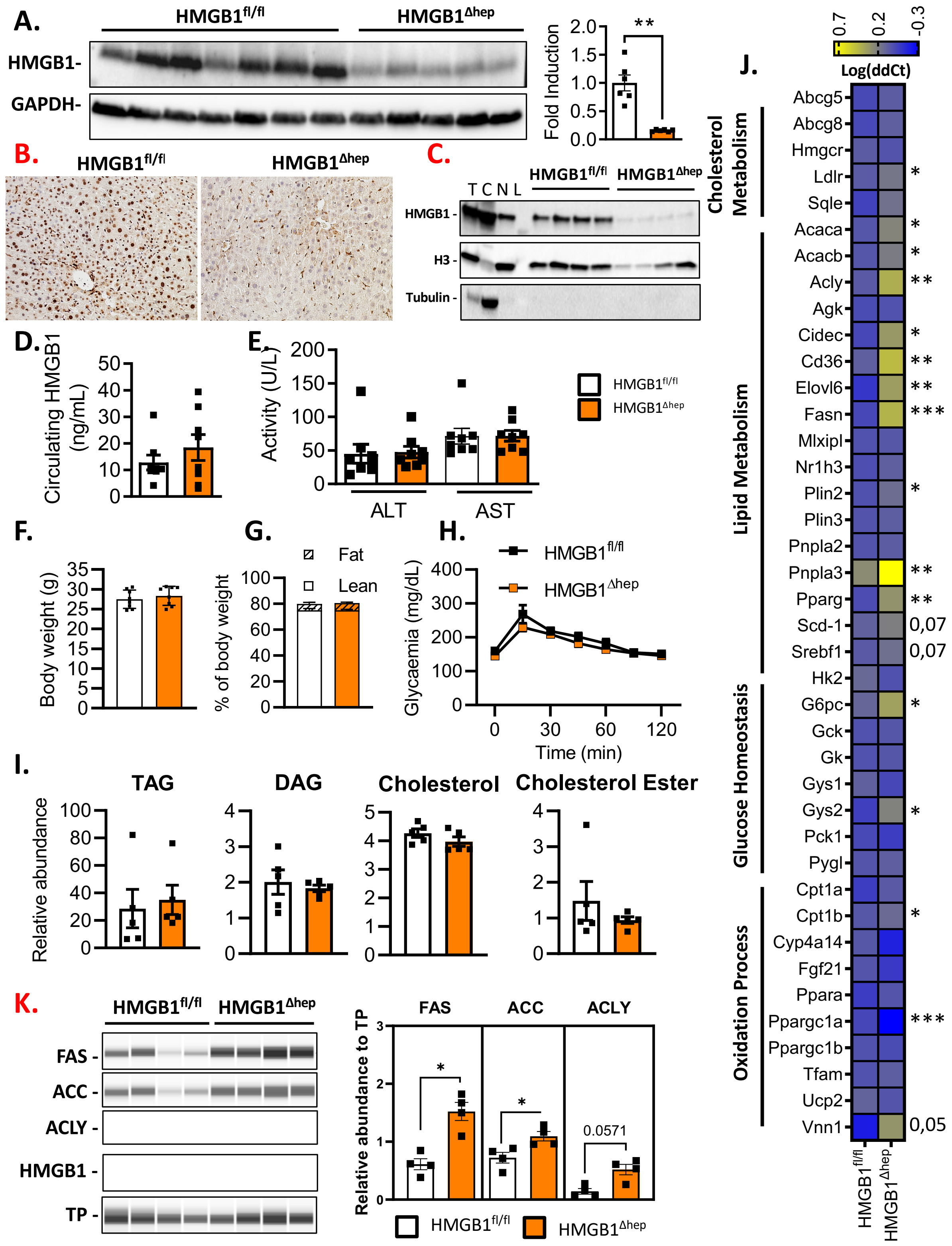
A.
HMGB1fl/fl
HMGB1Δhep
HMGB1-
GAPDH-
Fold Induction
**
B.
HMGB1fl/fl
HMGB1Δhep
C.
T C N L
HMGB1fl/fl
HMGB1Δhep
HMGB1 -
H3 -
Tubulin -
D.
Circulating HMGB1 (ng/mL)
E.
Activity (U/L)
ALT
AST
HMGB1fl/fl
HMGB1Δhep
F.
Body weight (g)
G.
Fat
Lean
% of body weight
H.
HMGB1fl/fl
HMGB1Δhep
Glycaemia (mg/dL)
Time (min)
I.
TAG
DAG
Cholesterol
Cholesterol Ester
Relative abundance
J.
Log(ddCt)
0.7
0.2
-0.3
Cholesterol Metabolism
Lipid Metabolism
Glucose Homeostasis
Oxidation Process
Abcg5
Abcg8
Hmgcr
Ldlr *
Sqle
Acaca *
Acacb *
Acly **
Agk
Cidec *
Cd36 **
Elovl6 **
Fasn ***
Mlxipl
Nr1h3
Plin2 *
Plin3
Pnpla2
Pnpla3 **
Pparg **
Scd-1 0,07
Srebf1 0,07
Hk2
G6pc *
Gck
Gk
Gys1
Gys2 *
Pck1
Pygl
Cpt1a
Cpt1b *
Cyp4a14
Fgf21
Ppara
Ppargc1a ***
Ppargc1b
Tfam
Ucp2
Vnn1 0,05
HMGB1fl/fl
HMGB1Δhep
K.
HMGB1fl/fl
HMGB1Δhep
FAS -
ACC -
ACLY -
HMGB1 -
TP -
FAS
ACC
ACLY
Relative abundance to TP
*
*
0.0571
HMGB1fl/fl
HMGB1Δhep

Fig. S1. Metabolic explorations of Hepatocyte specific *Hmgb1* deleted mice subjected to chow-diet.

(**A**) Representative immunoblot targeting HMGB1. GAPDH was used as a loading control. Densitometric quantification (right panel) performed on the whole animal cohort, on liver biopsies from on 8 week-old HMGB1$^{fl/fl}$ (n=7) and HMGB1$^{\Delta Hep}$ (n=5) mice fed on chow diet. (**B**) HMGB1 staining determined by IHC on liver sections and (**C**) immunoblot targeting HMGB1 on nuclear extracts in livers from HMGB1$^{fl/fl}$ and HMGB1$^{\Delta Hep}$ mice fed on chow diet (**C**). Histone 3 (H3) was used as a nuclear marker while Tubulin was used as a cytoplasm marker and control lanes as follow: T= total lysates, C=Cytoplasm extracts, N=Nucleus extracts and L=ladder. (**D-E**) Circulating levels of HMGB1 (**D**) and hepatic transaminases (**E**), (**F**) Body weight, (**G**) percentage of lean and fat mass, (**H**) analysis of oral glucose tolerance test and (**I**) liver neutral lipid levels in liver sections of 8 week-old HMGB1$^{fl/fl}$ and HMGB1$^{\Delta Hep}$ mice fed on chow diet. (**J**) Heatmap showing gene expression of specific markers involved in hepatic lipid and glucose metabolism determined by microfluidic RT-qPCR. (**K**) Immunoblot targeting key lipogenic enzymes FAS, ACC, ACLY and HMGB1 was performed on liver biopsies from on 12 week-old HMGB1$^{fl/fl}$ (n=4) and HMGB1$^{\Delta Hep}$ (n=4) mice fed on chow diet and densitometric quantification (right panel) normalized by the total protein load (TP). Data are means ± SEM of three independent experiments. *p<0.05, **p<0.01, ***p<0.001, ****p<0.0001 by unpaired Mann and Whitney comparison

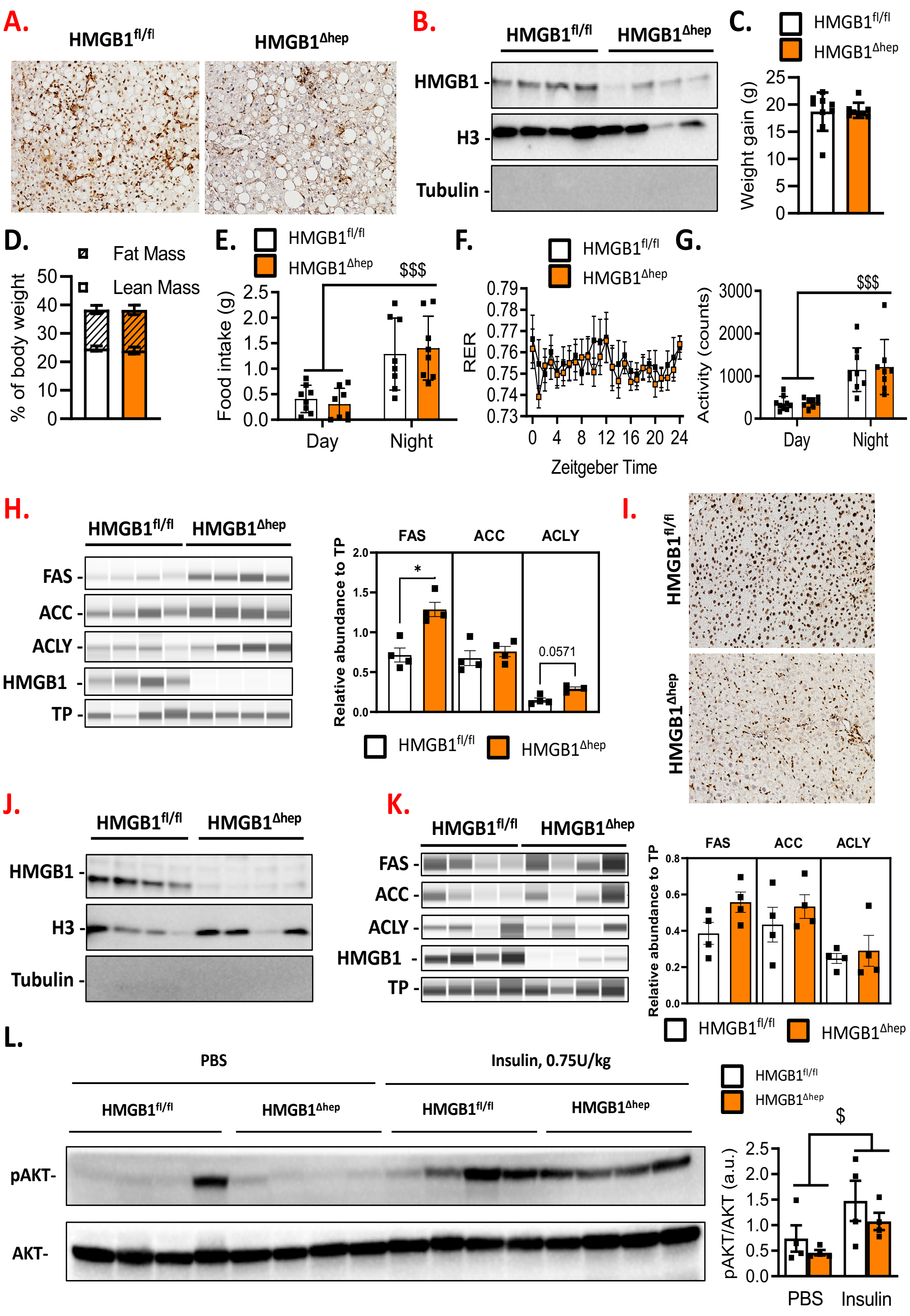

A.
HMGB1fl/fl
HMGB1Δhep
B.
HMGB1fl/fl
HMGB1Δhep
HMGB1 -
H3 -
Tubulin -
C.
HMGB1fl/fl
HMGB1Δhep
Weight gain (g)
D.
Fat Mass
Lean Mass
% of body weight
E.
HMGB1fl/fl
HMGB1Δhep
$$$
Food intake (g)
Day
Night
F.
HMGB1fl/fl
HMGB1Δhep
RER
Zeitgeber Time
G.
$$$
Activity (counts)
Day
Night
H.
HMGB1fl/fl
HMGB1Δhep
FAS -
ACC -
ACLY -
HMGB1 -
TP -
FAS
ACC
ACLY
Relative abundance to TP
*
0.0571
HMGB1fl/fl
HMGB1Δhep
I.
HMGB1fl/fl
HMGB1Δhep
J.
HMGB1fl/fl
HMGB1Δhep
HMGB1 -
H3 -
Tubulin -
K.
HMGB1fl/fl
HMGB1Δhep
FAS -
ACC -
ACLY -
HMGB1 -
TP -
FAS
ACC
ACLY
Relative abundance to TP
HMGB1fl/fl
HMGB1Δhep
L.
PBS
Insulin, 0.75U/kg
HMGB1fl/fl
HMGB1Δhep
HMGB1fl/fl
HMGB1Δhep
pAKT-
AKT-
HMGB1fl/fl
HMGB1Δhep
$
pAKT/AKT (a.u.)
PBS
Insulin

Fig. S2. Metabolic explorations of Hepatocyte specific *Hmgb1* deleted mice subjected to high-fat diet and F/R.

(**A-B**) HMGB1 staining determined by IHC on liver section (**A**) and immunoblot targeting HMGB1 on nuclear extracts from livers of $HMGB1^{fl/fl}$ and $HMGB1^{\Delta Hep}$ mice fed on 12 week-high-fat diet. Histone 3 (H3) was used as a nuclear marker while Tubulin was used as a cytoplasm marker (**B**). (**C**) Weight gain and (**D**) body fat and lean mass of $HMGB1^{fl/fl}$ (n=9) and $HMGB1^{\Delta Hep}$ (n=8) mice on HFD. (**E-G**) Indirect calorimetry analysis monitoring (**E**) food intake, (**F**) Respiratory exchange Ratio (RER) and (**G**) spontaneous physical activity of $HMGB1^{fl/fl}$ (n=8) and $HMGB1^{\Delta Hep}$ (n=8) mice on HFD during 24 hours. (**H**) Immunoblot targeting key lipogenic enzymes FAS, ACC, ACLY and HMGB1 was performed on liver biopsies from $HMGB1^{fl/fl}$ (n=4) and $HMGB1^{\Delta Hep}$ (n=4) mice fed subjected to12 week-HFD60% and densitometric quantification (right panel) normalized by the total protein load (TP). (**I-J**) HMGB1 staining determined by IHC on liver section (**I**) and immunoblot targeting HMGB1 on nuclear extracts from livers of $HMGB1^{fl/fl}$ and $HMGB1^{\Delta Hep}$ mice after F/r challenge (**J**). (**K**) Immunoblot targeting key lipogenic enzymes FAS, ACC, ACLY and HMGB1 was performed on liver biopsies from on 8 week-old $HMGB1^{fl/fl}$ (n=4) and $HMGB1^{\Delta Hep}$ (n=4) mice after F/R challenge and densitometric quantification (right panel) normalized by the total protein load (TP). **(L)** Representative immunoblot targeting p-AKT and Tot-AKT was used as a loading control. Densitometric quantification (right panel) performed on the whole animal cohort, on liver biopsies from on 8 week-old $HMGB1^{fl/fl}$ and $HMGB1^{\Delta Hep}$ mice fed on chow diet and challenged with an acute injection of PBS (100uL) (n=4 for each genotype) or insulin (i.p, 0.75 U/kg) (n=4 for each genotype). Livers were harvested 15 minutes after insulin stimulation. Data are means ± SEM of three independent experiments. *p<0.05, **p<0.01, ***p<0.001, ****p<0.0001 by unpaired Mann and Whitney comparison or two-way ANOVA. **$** p<0.05, **$$** p<0.01, **$$$** p<0.001, for treatment effect by two-way ANOVA.

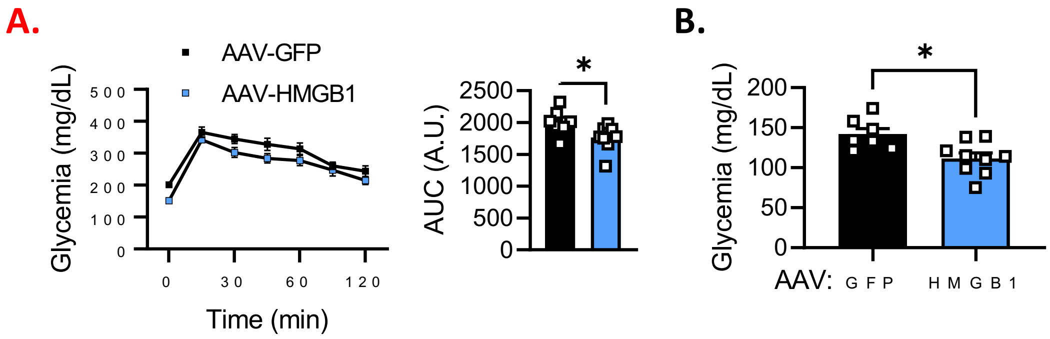

Fig. S3. Hepatic overexpression of HMGB1 improves glucose homeostasis.
(**A**) Analysis of oral glucose tolerance test with AUC representation and (**B**) fasting glycemia levels after overnight fasting. Data are means ± SEM of three independent experiments. *p<0.05, by unpaired Mann and Whitney comparison

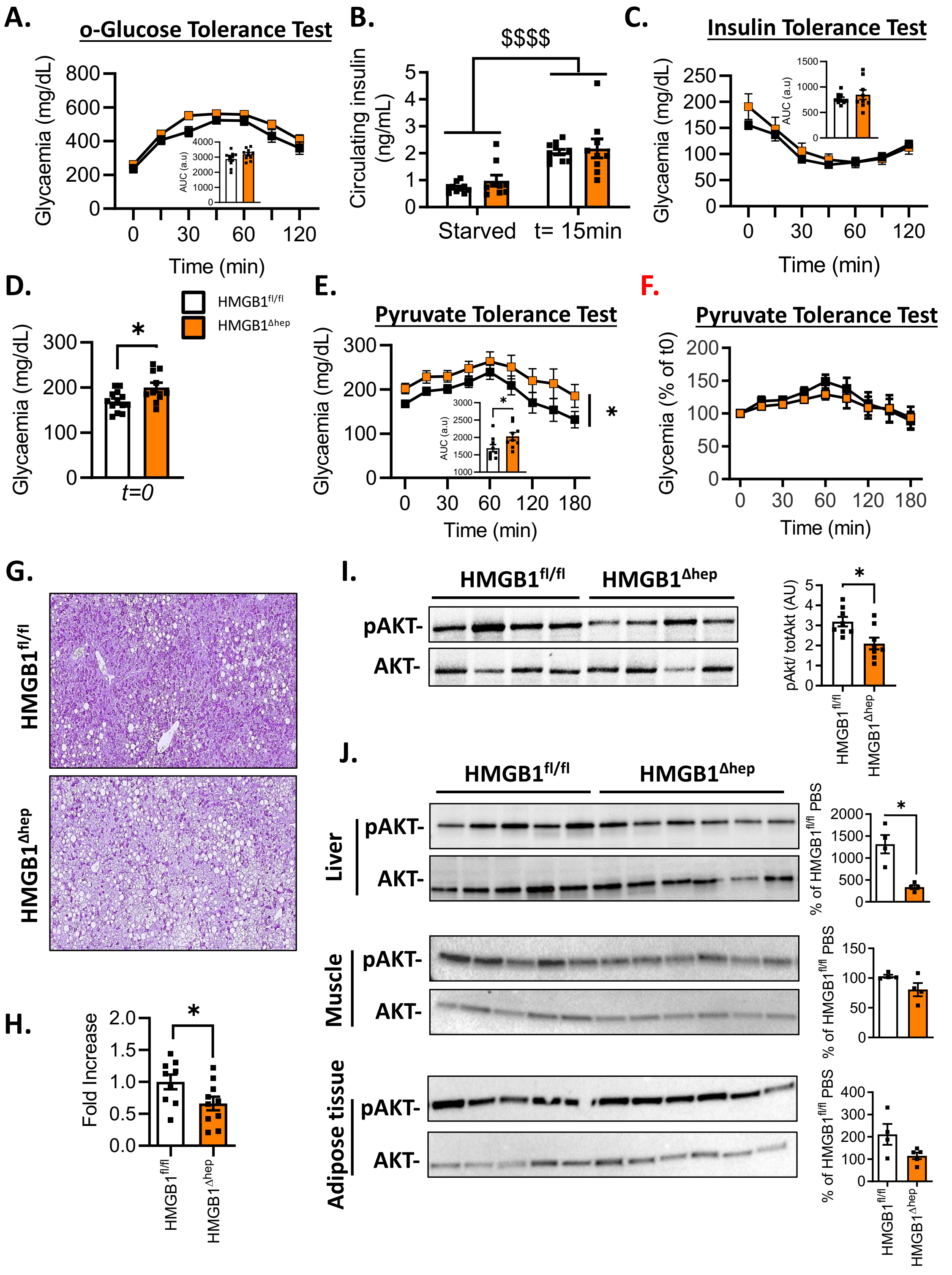
A.
o-Glucose Tolerance Test
Glycaemia (mg/dL)
Time (min)
AUC (a.u)
B.
$$$$
Circulating insulin (ng/mL)
Starved
t= 15min
C.
Insulin Tolerance Test
Glycaemia (mg/dL)
Time (min)
AUC (a.u)
D.
HMGB1fl/fl
HMGB1Δhep
Glycaemia (mg/dL)
t=0
E.
Pyruvate Tolerance Test
Glycaemia (mg/dL)
Time (min)
AUC (a.u)
F.
Pyruvate Tolerance Test
Glycemia (% of t0)
Time (min)
G.
HMGB1fl/fl
HMGB1Δhep
H.
Fold Increase
HMGB1fl/fl
HMGB1Δhep
I.
HMGB1fl/fl
HMGB1Δhep
pAKT-
AKT-
pAkt/ totAkt (AU)
J.
HMGB1fl/fl
HMGB1Δhep
Liver
Muscle
Adipose tissue
pAKT-
AKT-
% of HMGB1fl/fl PBS

Fig. S4. Hepatocyte specific *Hmgb1* deleted mice on HFD display reduced insulin sensitivity in the liver.

(**A**) Analysis of oral glucose tolerance test, (**B**) Insulin levels after fasting or 15 minutes post glucose bolus, (**C**) insulin tolerance test, (**D**) fasting glycaemia levels after 16 hours of fasting and (**E-F**) pyruvate tolerance test on $HMGB1^{fl/fl}$ and $HMGB1^{\Delta Hep}$ mice fed on HFD for 12 weeks expressed with absolute values (**E**) or percentage from the baseline (**F**). (**G-H**) Hepatic PAS staining representative images with (**H**) quantification on $HMGB1^{fl/fl}$ and $HMGB1^{\Delta Hep}$ mice fed on HFD for 12 weeks. (**I**) Representative immunoblot targeting p-AKT and tot-AKT with quantification performed on the whole animal cohort, on liver biopsies from on $HMGB1^{fl/fl}$ and $HMGB1^{\Delta Hep}$ mice fed on HFD for 12 weeks. (**J**) Representative immunoblot targeting p-AKT and tot-AKT with quantification performed on the whole animal cohort, on liver, skeletal muscle (gastrocnemius) and adipose tissue (perigonadal fat pad noted PG) biopsies from $HMGB1^{fl/fl}$ and $HMGB1^{\Delta Hep}$ mice fed on HFD for 24 weeks, starved 4 hours and injected with insulin (i.p. 0.75U/kg-15 minutes). Data are means ± SEM from n=10 ($HMGB1^{fl/fl}$) or n=11 ($HMGB1^{\Delta Hep}$) per group for the HFD protocol (**A-H**) and from n=4 ($HMGB1^{fl/fl}$) or n=4 ($HMGB1^{\Delta Hep}$) per group for the HFD 24-week with acute injection of insulin protocol (**J**). *p<0.05, **p<0.01, ***p<0.001, ****p<0,0001 by unpaired Mann and Whitney comparison or two-way ANOVA.

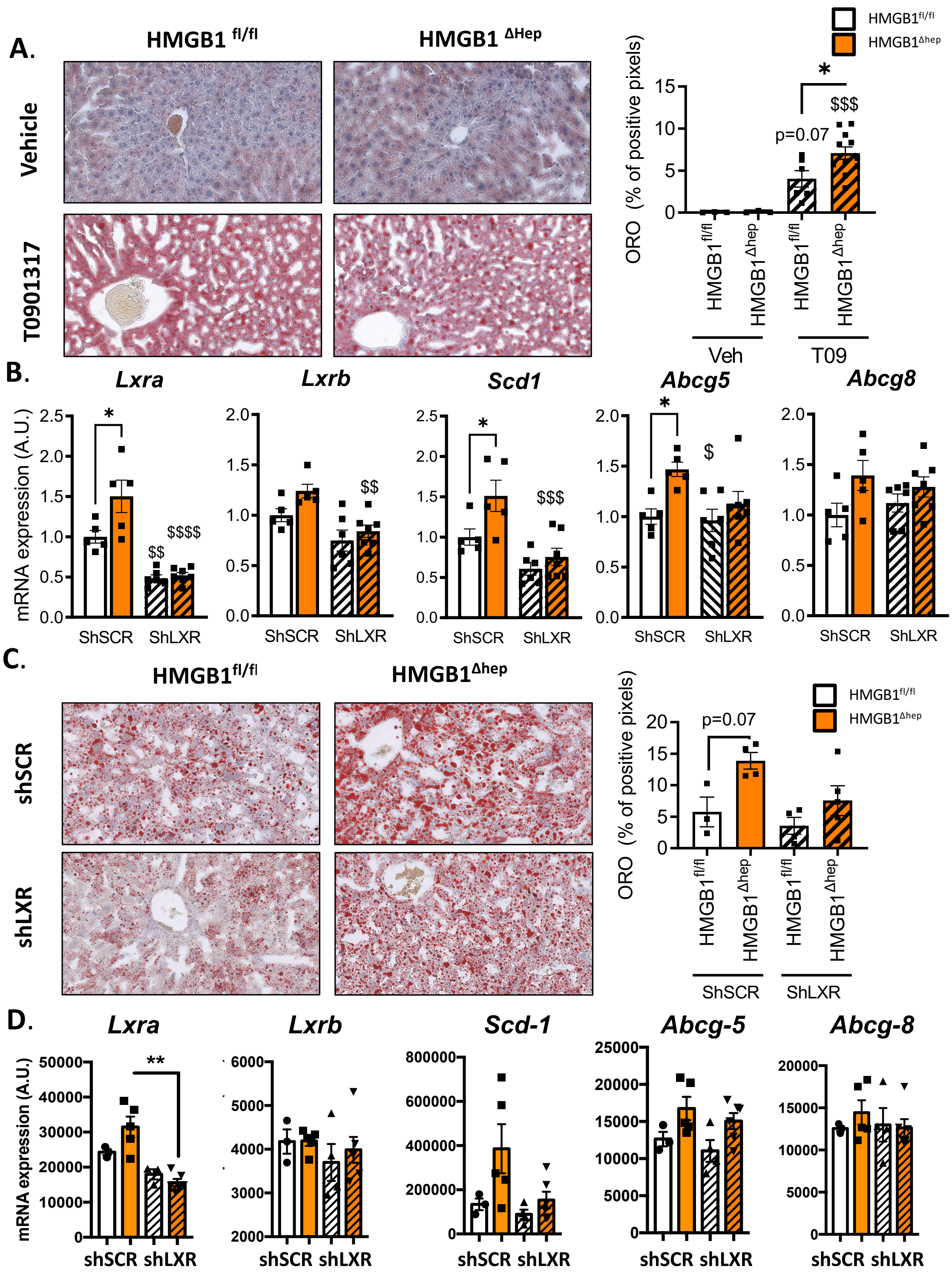
A.
HMGB1 fl/fl
HMGB1 ΔHep
Vehicle
T0901317
HMGB1fl/fl
HMGB1Δhep
ORO (% of positive pixels)
p=0.07
$$$
*
Veh
T09
B.
Lxra
Lxrb
Scd1
Abcg5
Abcg8
mRNA expression (A.U.)
$$
$$$$
$$$
$
ShSCR
ShLXR
C.
HMGB1fl/fl
HMGB1Δhep
shSCR
shLXR
p=0.07
D.
Lxra
Lxrb
Scd-1
Abcg-5
Abcg-8
**
shSCR shLXR

Fig. S5. Hepatocyte specific *Hmgb1* deleted mice exhibit a severe liver steatosis under metabolic stress that is restored by knocking-down LXRα *in vivo*.

(**A**) 8 week-old HMGB1$^{fl/fl}$ (n=5) and HMGB1$^{\Delta Hep}$ (n=9) mice fed on chow diet were treated either with vehicle (5% carboxy-methyl-cellulose) (HMGB1$^{fl/fl}$ (n=5) and HMGB1$^{\Delta Hep}$ (n=3)) or LXRα synthetic agonist T0901317 (oral gavage, 30 mg/kg/day) (HMGB1$^{fl/fl}$ (n=10) and HMGB1$^{\Delta Hep}$ (n=7)) for four consecutive days, after 6 hours starvation on the last day mice were sacrificed. (**A**) Liver steatosis was assessed by Oil Red-O staining on liver sections with the quantitative representation displayed on the right. (**B**) HMGB1$^{fl/fl}$ and HMGB1$^{\Delta Hep}$ mice were infected with either adenovirus expressing a scramble (shSCR, HMGB1$^{fl/fl}$, n=6 and HMGB1$^{\Delta Hep}$, n=7) or an LXRα shRNA (shLXR, HMGB1$^{fl/fl}$, n=5 and HMGB1$^{\Delta Hep}$, n=5) sequence, then subjected 7 days later to a F/R challenge was then subjected to RT-qPCR analysis of the indicated LXRα dependent genes. (**C-D**) HMGB1$^{fl/fl}$ and HMGB1$^{\Delta Hep}$ mice were subjected to HFD for four weeks and then infected with either adenovirus expressing a scramble (shSCR, HMGB1$^{fl/fl}$, n=2 and HMGB1$^{\Delta Hep}$, n=5) or an *Lxrα* shRNA (sh*Lxrα*, HMGB1$^{fl/fl}$, n=4 and HMGB1$^{\Delta Hep}$, n=6) sequence and euthanized 7 days later. Liver steatosis was determined by quantifying Oil Red-O staining (**C**) and sh*Lxrα* efficiency was assessed by measuring indicated LXRα dependent gene expression using RT-qPCR (**D**). Data are means ± SEM. *p<0.05, **p<0.01, ***p<0.001, ****p<0.0001 for HMGB1$^{fl/fl}$ and HMGB1$^{\Delta Hep}$ comparison, by unpaired Mann and Whitney comparison. $ p<0.05, $$ p<0.01, $$$ p<0.001, for treatment effect by one-way ANOVA.

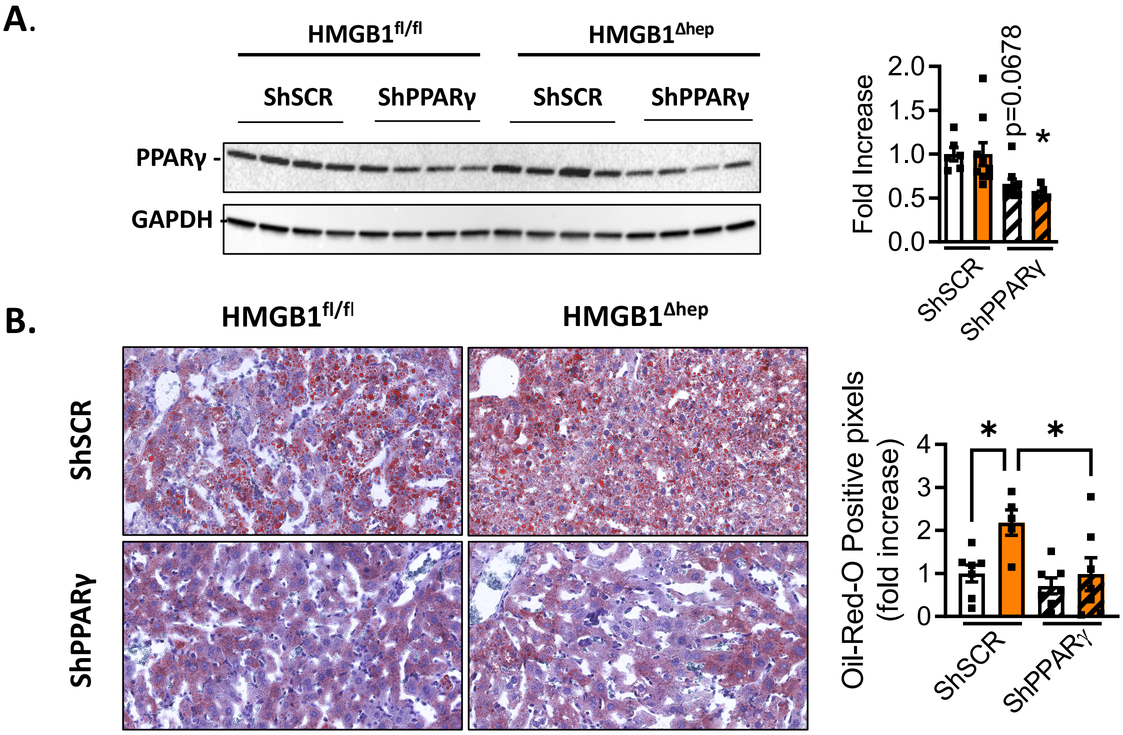

Fig. S6. Hepatocyte specific *Hmgb1* deleted mice exhibit a severe liver steatosis under metabolic stress that is restored by knocking-down PPARγ *in vivo*.

(**A**) Immunoblot targeting PPARγ, with GAPDH used as a loading control. (**B**) $HMGB1^{fl/fl}$ and $HMGB1^{\Delta Hep}$ mice were subjected to HFD for four weeks and then infected with either adenovirus expressing a PPARγ shRNA (shPPARγ, $HMGB1^{fl/fl}$, n=7 and $HMGB1^{\Delta Hep}$, n=7)) or a scramble (shSCR, $HMGB1^{fl/fl}$, n=7 and $HMGB1^{\Delta Hep}$, n=5) sequence and euthanized 7 days later. Liver steatosis was assessed by Oil Red-O staining on liver section tissue with the quantitative representation displayed on the right. *p<0.05, **p<0.01, ***p<0.001, ****p<0.0001 for $HMGB1^{fl/fl}$ and $HMGB1^{\Delta Hep}$ comparison, by unpaired Mann and Whitney comparison or by one-way ANOVA.

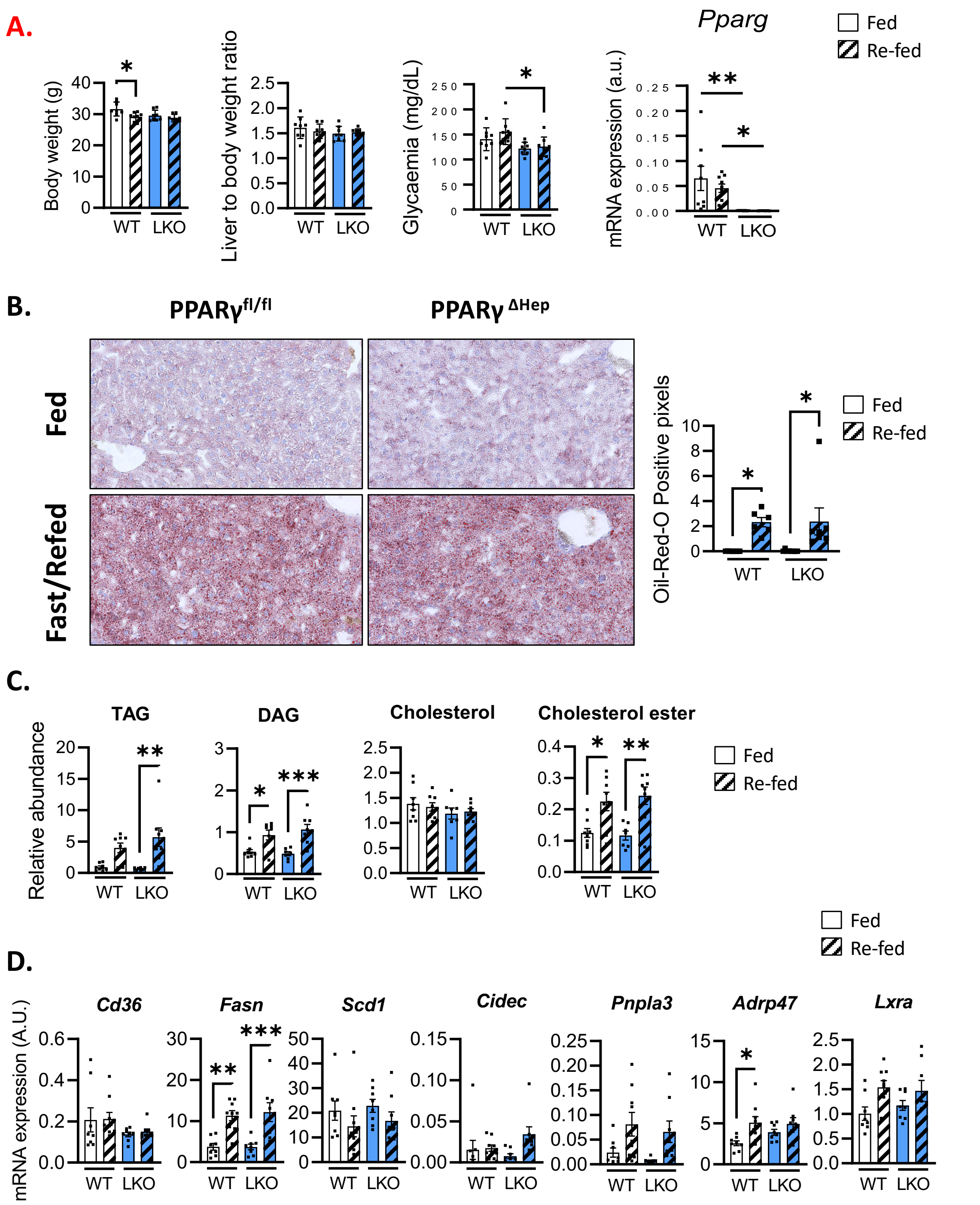

Fig. S7. Hepatocyte specific *Pparγ* deletion does not modify liver steatosis in mice after F/R challenge.

PPAR*γ* fl/fl and PPAR*γ* ΔHep (n=8) mice were subjected to F/R challenge. (**A**) Body weight, liver/body weight ratio and starved glycaemia of mice on fed (6 hours)-refeed (8 hours). Hepatic steatosis was assessed by (**B**) Oil Red-O staining with quantification (right panel) and (**C**) neutral lipids analysis. (**D**) mRNA expression of hepatic steatosis markers from liver biopsies was determined by RT-qPCR. Data are means ± SEM of three independent experiments. *p<0.05, **p<0,01, ***p<0.001, ****p<0.0001 by unpaired Mann and Whitney comparison.

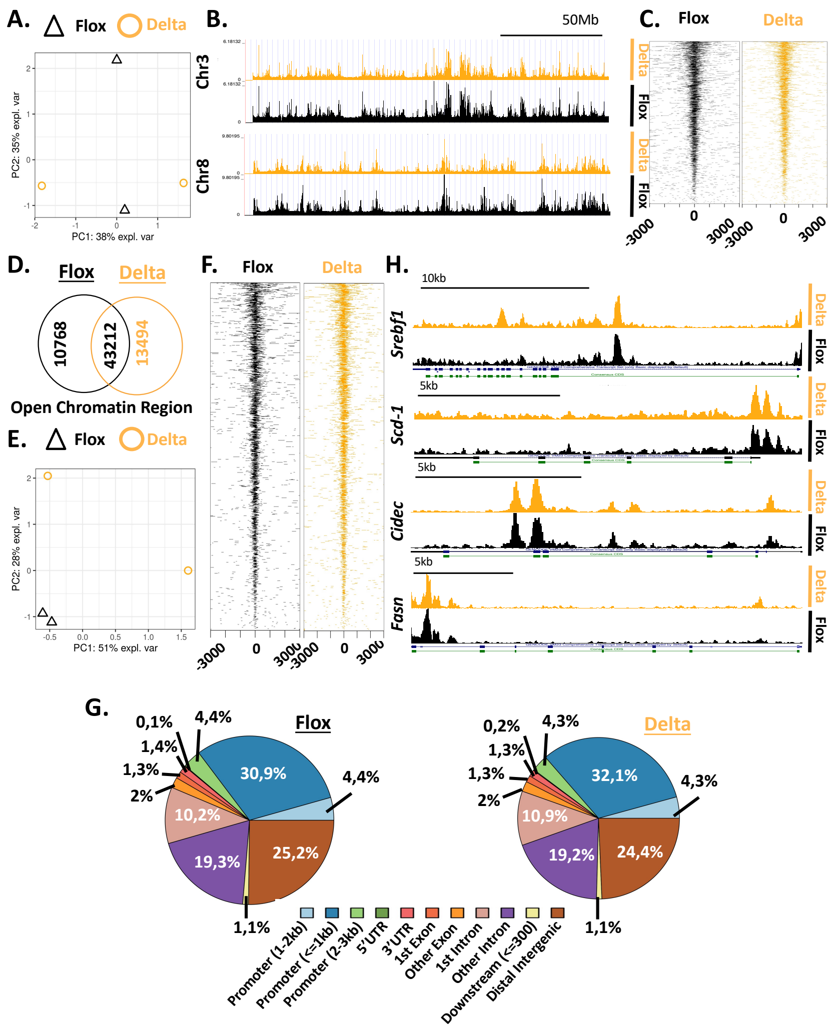

Fig. S8. Hepatocyte specific *Hmgb1* deletion does not remodel chromatin.
(**A**) Principal component analysis scores plot of ATAC-seq data of liver tissue from HMGB1$^{fl/fl}$ (black triangle, n=2) and HMGB1$^{\Delta Hep}$ (orange circle, n=2)) mice on chow diet. (**B**) UCSC genome browser of tracks showing HMGB1 similar chromatin open region in Chromosome 3 and 8. (**C**) Average coverage around TSS in liver biopsies of HMGB1$^{fl/fl}$ (black) and HMGB1$^{\Delta Hep}$ (orange) mice. (**D**) Venn Diagram showing overlapping opened region and and (**E**) Principal component analysis scores plot of ATAC-seq data of liver tissue from HMGB1$^{fl/fl}$ (black triangle, n=2)) and HMGB1$^{\Delta Hep}$ (orange circle, n=2) mice subjected to F/R. (**F**) Average coverage around TSS in liver biopsies of HMGB1$^{fl/fl}$ (black) and HMGB1$^{\Delta Hep}$ (orange) mice after F/R challenge. (**G**) Chart pie displaying the genomic distribution of open chromatin domain in liver biopsies from HMGB1$^{fl/fl}$ (black, n=2)) and HMGB1$^{\Delta Hep}$ (orange, n=2) mice. (**H**) UCSC genome browser snapshots of tracks showing HMGB1 chromatin open region of canonical LXRα and PPARγ responsive gene loci (*Srebf1*, *Scd-1*, *Cidec*, *Fasn*) in liver biopsies of HMGB1$^{fl/fl}$ (black) and HMGB1$^{\Delta Hep}$ (orange) mice after F/R challenge.

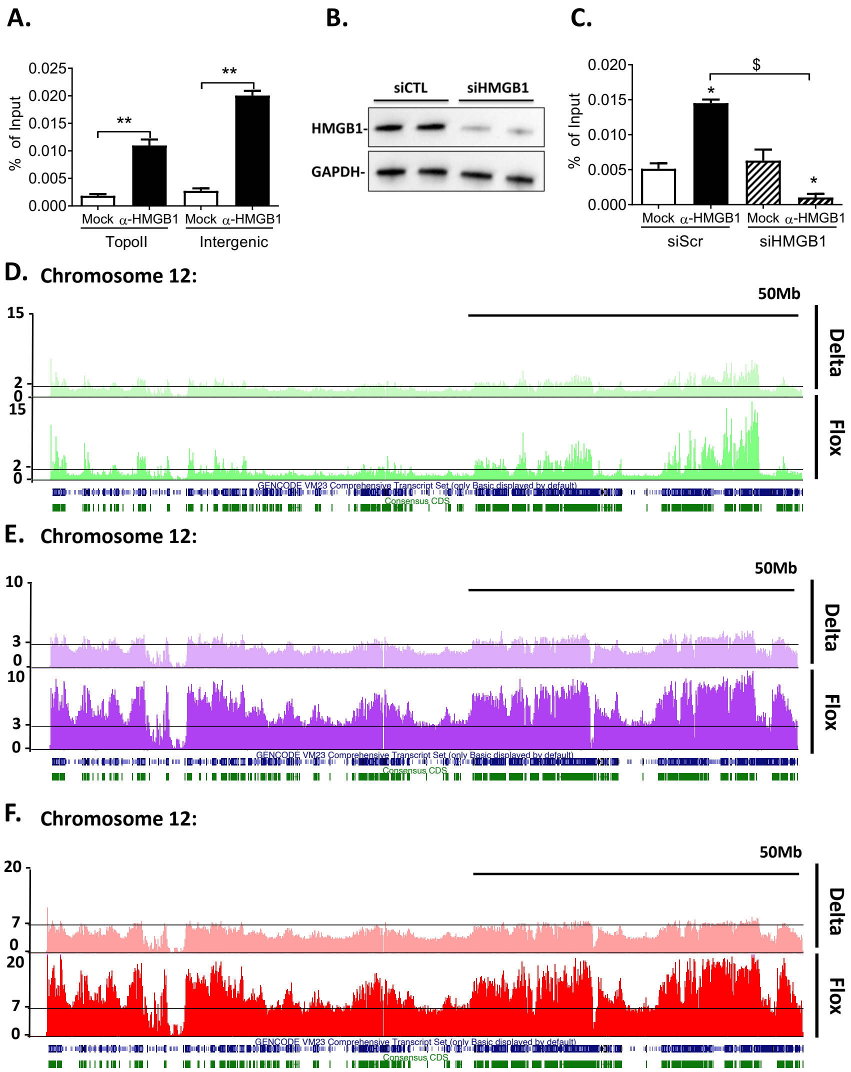

Fig. S9. Validation of HMGB1 ChIP *in vitro* and *in vivo*.

(**A**) Chromatin immuno-precipitation was performed against HMGB1 on U2OS cells and enrichment on TOPO isomerase II promoter sequence as well as intergenic region were measured by RT-qPCR. (**B-C**) To validate specificity of the ChIP, (**B**) U2OS cells were first transfected with a siRNA targeting HMGB1 and after successful knockdown of HMGB1 showed by immunoblot targeting HMGB1 with GAPDH as a loading control, (**C**) ChIP experiment has been conducted, and siHMGB1 treated cells showed no DNA enrichment of TOPOII promoter region while siScramble exhibits a significant DNA enrichment. (**D-F**) UCSC genome browser of tracks from the ChIP-seq data, comparing the sequencing signal in Chromosome 2 from HMGB1$^{fl/fl}$ (n=2) and HMGB1$^{\Delta Hep}$ (n=2) mice upon (**D**) Chow diet (green), (**E**) HFD (purple) and (**F**) F/R (red). Data are means ± SEM of three independent experiments (**A-C**) *p<0.05 ** p<0.01 αHMGB1 vs Mock, **$**<0.05 siScramble vs siHMGB1 by unpaired Mann and Whitney comparison or two-way ANOVA

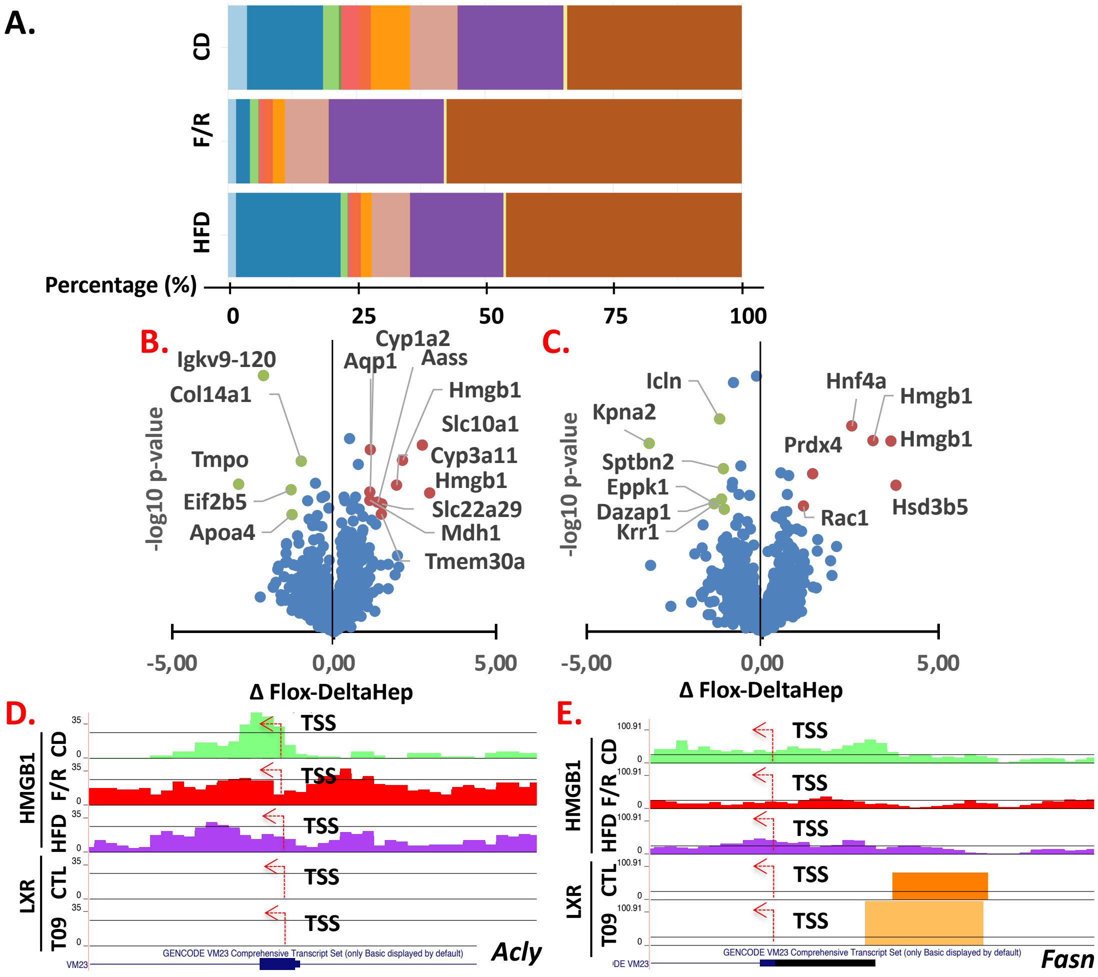

F.

Motif (de novo)	TF (TomTom)	E-Value
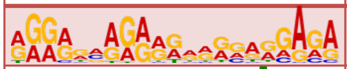	ZNF263	1.1e-096
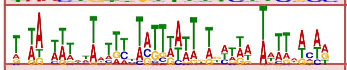	0 match	2.4e-062
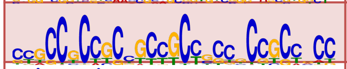	SP2-ZNF263-SP1	4.1e-065
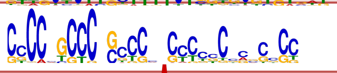	SP2-ZNF263-RREB1-SP1-KLF5-SP3-SP8-KLF16-ZNF140-EGR4-ZIC4-ZIC3-THAP1-KLF9-ZFX-MZF-1	3.6e-029
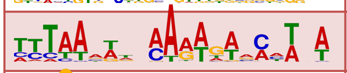	0 match	2.9e-010
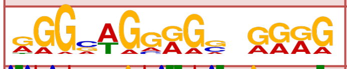	ZNF263-KLF5-SP2-SP1-E2F6-INSM1-SP3	6.3e-011
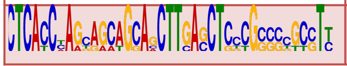	0 match	58e-003

Fig. S10. Genomic distribution and proteomic analyses of HMGB1.

(**A**) Genomic distributions of enriched regions identified in ChIP-seq data sets in liver biopsies of HMGB1$^{fl/fl}$ mice subjected to chow diet, HFD and F/R. (**B-C**) Volcano plots of proteins identified to be associated with HMGB1 in liver nuclear extracts of mice (n=4) subjected to HFD (**B**) or after F/R (**C**). (**D-E**) UCSC genome browser shot of HMGB1 and LXRα ChIP-seq data along the locus of *Acly* (**D**) and *Fasn* (**E**) gene loci. HMGB1 tracks in liver from HMGB1$^{fl/fl}$ upon chow diet (green), after F/R (red) and HFD (purple). LXRα tracks from basal liver (dark orange) and T09 challenged liver (light orange). Coverage on gene locus and TSS region (-1kb/+1kb) of LXRα responsive gene: *Acly* (**D**) and *Fasn* (**E**). Exon 1 model (blue) is displayed on the bottom track. (**F**) Table of significant *de novo* motifs identified, using the MEME Suite, across the HMGB1 ChIP peaks in the promoter region (+/-3kb) flanking the TSS of 134 genes belonging to the metabolism GO term.

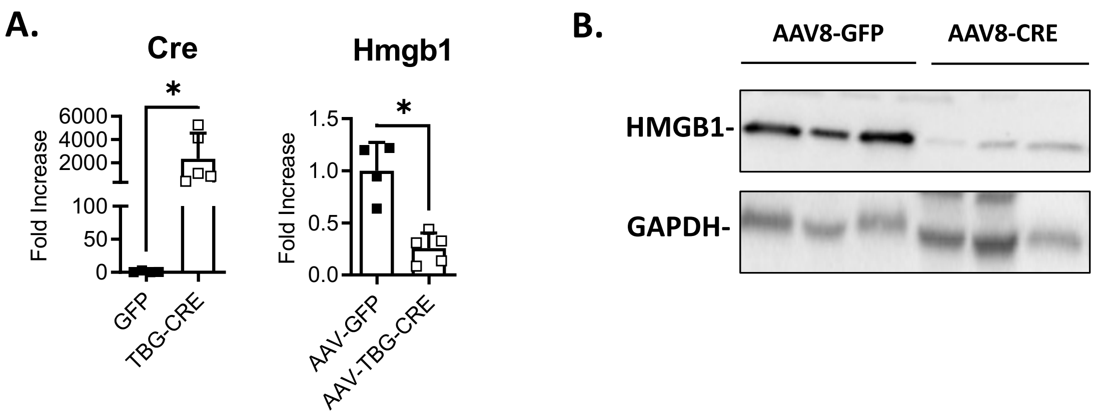

Fig. S11. Successful *in vivo* knockdown of *Hmgb1* gene and protein using AAV-TBG-Cre.

Adult HMGB1$^{fl/fl}$ mice were infected either with AAV8-Gfp (n=3) or AAV8-Tbg-Cre (n=3) to selectively generate *Hmgb1* deletion in hepatocytes *in vivo.* (**A**) The level of expression of *Hmgb1* gene was determined using RT-qPCR in whole liver. (**B**) Representative immunoblot targeting HMGB1 in liver extracts. GAPDH was used as a loading control. Data are means ± SEM. *p<0.05, **p<0.01, ***p<0.001, ****p<0.0001 by unpaired Mann and Whitney comparison.

Table S1. List of genes highly occupied by HMGB1 in Chow Diet compare to HFD

GO « Integration of Energy Metabolism »	GO « Phospholipid Metabolism »
Abcc8	Acp6
Acaca	Agpat1
Acacb	Agpat5
Acly	Arf1
Acsl3	Awat2
Adcy5	Cds2
Adipor2	Cept1
Adra2c	Cpne1
Cacna1a	Cpne3
Cacna1c	Cpne7
Cacna2d2	Ddhd2
Cacnb2	Enpp6
CD36	Etnk1
Fasn	Gnpat
Gcg	Gpat2
Gcgr	Gpat3
Glp1r	Gpat4
Gna11	Hadha
Gna14	Inpp4b
Gna15	Inpp5f
Gnai1	Liph
Gnai2	Lpcat2
Gnaq	Lpcat3
Gnas	Lpin3
Gnb1	Mfsd2a
Gnb2	Mgll
Gnb3	Miga1
Gng11	Mtm1
Gng12	Mtmr14
Gng13	Mtmr7
Gng3	Osbpl10
Gng5	Osbpl5
Gng8	Osbpl8
Itpr1	Phospho1
Itpr2	Pik3ca
Itpr3	Pik3cd
Kcnb1	Pik3r2
Kcnc2	Pik3r3
Kcng2	Pip5k1a
Kcnj11	Pip5k1b
Marcks	Pitpnb
Mlx	Pitpnm2

Mlxipl	Pla2g12a
Plcb1	Pla2g15
Plcb2	Pla2g1b
Plcb3	Pla2g2d
Prkag2	Pla2g2e
Prkar1a	Pla2g4d
Prkar1b	Pla2g4f
Prkca	Pla2g5
Rapgef4	Plb1
Slc2a1	Plekha1
	Plekha2
	Plekha5
	Pnpla6
	Pnpla8
	Ptpn13
	Rab4a
	Selenoi
	Slc44a1
	Slc44a2
	Slc44a3
	Slc44a5
	Stard10
	Stard7
	Taz
	Tnfaip8l1
	Tnfaip8l2
	Tnfaip8l3
	Tpte
	Vac14

Table S2. List of genes highly occupied by HMGB1 in Chow Diet compare to F/R

GO « Integration of Energy Metabolism »	GO « Phospholipid Metabolism »
Acaca	Acp6
Acacb	Agpat1
Acly	Agpat5
Acsl3	Arf1
Acsl4	Awat2
Adcy5	Cds2
Adcy6	Cept1
Adipor2	Cpne1
Adra2c	Cpne3
Agpat1	Cpne7
Cacna1a	Ddhd2
Cacna1c	Enpp6
Cacna2d2	Etnk1
Cacnb2	Gnpat
Cacnb3	Gpat2
Cd36	Gpat3
Fasn	Gpat4
Gcg	Hadha
Gcgr	Inpp4b
Glp1r	Inpp5f
Gna11	Liph
Gna14	Lpcat2
Gna15	Lpcat3
Gnai1	Lpin3
Gnai2	Mfsd2a
Gnaq	Mgll
Gnas	Miga1
Gnb1	Mtm1
Gnb2	Mtmr14
Gnb3	Mtmr7
Gng10	Osbpl10
Gng11	Osbpl5
Gng12	Osbpl8
Gng13	Phospho1
Gng3	Pik3ca
Gng5	Pik3cd
Itpr1	Pik3r2
Itpr2	Pik3r3
Itpr3	Pip5k1a
Kcnb1	Pip5k1b
Kcnc2	Pitpnb
Kcng2	Pitpnm2
Kcns3	Pla2g12a
Marcks	Pla2g15
Mlx	Pla2g1b

Mlxipl	Pla2g2d
Plcb1	Pla2g2e
Plcb2	Pla2g4d
Plcb3	Pla2g4e
Prkab2	Pla2g4f
Prkaca	Pla2g5
Prkag2	Plb1
Prkar1a	Plekha1
Prkar1b	Plekha2
Prkar2b	Plekha5
Prkca	Pnpla6
Rap1a	Pnpla8
Rapgef3	Ptpn13
Rapgef4	Rab4a
Slc2a1	Selenoi
	Slc44a1
	Slc44a2
	Slc44a3
	Slc44a5
	Stard10
	Stard7
	Taz
	Tnfaip8l1
	Tnfaip8l2
	Tnfaip8l3
	Tpte
	Vac14

Table S3. Primers for Real time qPCR

Gene	Forward Primer	Reverse Primer
36B4	AGTCGGAGGAATCAGATGACGAT	GGCTGACTTGGTTGCTTTGG
Abcg5	TCGCAACGGTCATTTTCA	GCCAAAAGAGCAGCAGAGAAATA
Abcg8	GCGTCTGTCGATGCTGGTC	ATCCATTGGCCACCCTTGT
Acc1	CTCCTTTGCCTTCCGACATC	TACCATGCCAATCTCATTTCCTC

Acc2	TGGAGGCAACAGGGTCATAGA	CGCAGCGATGCCATTGT
Acly	AAAGCTTGGCCTCGTCGG	GGGACGAAGGGTTCAATGAGA
Adrp47	GTGTCGTCGTAGCCGATGC	CCATTTCTCAGCTCCACTCCAC
Agk	GTGTTTGGCAACCAGCTCATT	GCTGCGGGATTGAGAAAGAC
Fabp4	TTCGATGAAATCACCGCAGA	GGTCGACTTTCCATCCCACTT
Cd11b	TCGGACGAGTTCCGGATTC	TGTGATCTTGGGCTAGGGTTTC
Cd11c	GATTTCAGCATCCCAGATCCC	CCAGATCCACCAGTCCATCC
Cd36	GGACATACTTAGATGTGGAACCCATA	TGTTGACCTGCAGTCGTTTTG
Cd45	ACATGCTGCCAATGGTTCTG	GTCCCACATGACTCCTTTCCTATG
Chrebp	ACATCAGCGCTTTGACCAGAT	TGCGCGTCCGGACATAG
Cidec	GACTTTATTGGCTGCCTGAACG	ATCTCCTTCACGATGCGCTT
Cpt1a	CACCAACGGGCTCATCTTCT	CCTTCTATCGAATTTGCTCTGGTT
Cpt1b	GTGCAAGCAGCCCGTCTAG	TTGCGGCGATACATGATCA
Cre	CATTGCTGTCACTTGGTCGT	CATTTGGGCCAGCTAAACAT
Cyp4a14	TCAGTCTATTTCTGGTGCTGTTC	GAGCTCGTTGTCCTTCAGATGGT
Elovl6	TCTGATGAACAAGCGAGCCA	TGGTCATCAGAATGTACAGCATGT
F4/80	TGACAACCAGACGGCTTGTG	GCAGGCGAGGAAAAGATAGTGT
Fasn	ATCCTGGAACGAGAACACGATCT	AGAGACGTGTCACTCCTGGACTT
Fgf21	GGTCAAGTCCGGCAGAGGTA	CTGTTCCATCCTCCCTGATCTC
G6pc	ACGTATGGATTCCGGTGTTTG	CAGCTGCACAGCCCAGAA
Gck	TCGCAGGTGGAGAGCGA	TCGCAGTCGGCGACAGA
Gk	GTGTTTGGCAACCAGCTCATT	GCTGCGGGATTGAGAAAGAC
Gys1	CGGCTTTGGCTGCTTTATG	CCAGAATGTAAATGCCGTAAGCT
Gys2	CCAACGACGGATGGCTTTAA	GATCCTGACGGAGAAGGTGGTA
Hk2	CGCCGGATTGGAACAGAA	CCCGTCGCTAACTTCACTCACT
Hmgb1	AGCCCTGTCCTGGTGGTATT	CCAGGCAAGGTTAGTGGCTA
Hmgcr	TTGTTCACGCTCATAGTCGCT	GACACATCTTCGTCCAGACCC
L-pk	AGTCGTGCAATGTTCATCCCT	TCGACTCAGAGCCTGTGGC
Ldl-r	TGGATCCACCGCAACATCTA	CTCTTTACGCCCTTGGTGTCA
Lpl	TTATCCCAATGGAGGCACTTTC	CACGTCTCCGAGTCCTCTCTCT
Lxra	AGGAGTGTCGACTTCGCAAA	CTCTTCTTGCCGCTTCAGTTT
Lxrbeta	AAGCAGGTGCCAGGGTTCT	TGCATTCTGTCTCGTGGTTGT
Pck1	ATGTTCGGGCGGATTGAAG	TCAGGTTCAAGGCGTTTTCC
Pnpla2	CGCCTCTCGAAGGCTCTCTT	TGTAGCCCTGTTTGCACATCTC
Pnpla3	AGCCCGTCTCTGATGCACTT	ACGCGGTCACCTTCGTGT
Ppara	TGGCAGCAATATCAGAGGTAGATTC	ATCATATCAAAGGAGCTGCCAAA
Pparg (Fluidigm)<br>*Pparg (LKO)*	CCGAAGAACCATCCGATTGA<br>GATGCACTGCCTATGAGCACTT	TTTGTGGATCCGGCAGTTAAG<br>GAATGGCATCTCTGTGTCAACC
Pparg1ca	AAAGGATGCGCTCTCGTTCA	GGAATATGGTGATCGGGAACA
Ppargc1b	TTGAGGTGTTCGGTGAGATTGTAG	GAAGGTGATAAAACCGTGCTTCTG
Pygl	CAAGTGTCCCAAGAGGGTGT	TGTAATGTTCGGCCCATGTA
Scd-1	CAACACCATGGCGTTCCA	GGTGGGCGCGGTGAT

Serpin2	CAACACAGGGATCCAGGTCT	CATGAGGCCGTGACTTGATG
Sqle	CTGCACTTGGTTGGTTTCTGAC	GGAGGCTACCGTGTTCTCCA
Srebf1	CTGGCTTGGTGATGCTATGTTG	GACCATCAAGGCCCCTCAA
Tfam	GCACCCTGCAGAGTGTTCAA	CGCCCAGGCCTCTACCTT
Tip47	GGCTGGACAGACTGCAGGA	TCTTGAGCCCCAGACACTGTAG
Ucp2	CCTCAAAGCAGCCTCCAGAA	TCAATCGGCAAGACGAGACA
Vnn1	ATGAGGTTTATGCCTTTGGAGC	CCACAGGTGCGTAAATTGGTAG
